# Using genetic drug-target networks to develop new drug hypotheses for major depressive disorder

**DOI:** 10.1038/s41398-019-0451-4

**Published:** 2019-03-15

**Authors:** Héléna A. Gaspar, Zachary Gerring, Christopher Hübel, Major Depressive Disorder Working Group of the Psychiatric Genomics Consortium, Christel M. Middeldorp, Eske M. Derks, Gerome Breen

**Affiliations:** 1 King’s College London, Institute of Psychiatry, Psychology and Neuroscience, Social, Genetic and Developmental Psychiatry (SGDP) Centre, London, SE5 8AF UK; 2 National Institute for Health Research Biomedical Research Centre, South London and Maudsley National Health Service Trust, London, EC1V 2PD UK; 3 Translational Neurogenomics Laboratory, QIMR Berghofer Institute of Medical Research, Brisbane City, QLD 4006 Australia; 4 Department of Medical Epidemiology and Biostatistics, Karolinska Institutet, Stockholm, Sweden; 5 Child Health Research Centre, University of Queensland, South Brisbane, QLD 4072 Australia; 6 Child and Youth Mental Health Service, Children’s Health Queensland Hospital and Health Service, South Brisbane, QLD 4101 Australia; 7 Biological Psychology, Vrije Universiteit Amsterdam, 1081 HV Amsterdam, Netherlands

## Abstract

The major depressive disorder (MDD) working group of the Psychiatric Genomics Consortium (PGC) has published a genome-wide association study (GWAS) for MDD in 130,664 cases, identifying 44 risk variants. We used these results to investigate potential drug targets and repurposing opportunities. We built easily interpretable bipartite drug-target networks integrating interactions between drugs and their targets, genome-wide association statistics, and genetically predicted expression levels in different tissues, using the online tool Drug Targetor (drugtargetor.com). We also investigated drug-target relationships that could be impacting MDD. MAGMA was used to perform pathway analyses and S-PrediXcan to investigate the directionality of tissue-specific expression levels in patients vs. controls. Outside the major histocompatibility complex (MHC) region, 153 protein-coding genes are significantly associated with MDD in MAGMA after multiple testing correction; among these, five are predicted to be down or upregulated in brain regions and 24 are known druggable genes. Several drug classes were significantly enriched, including monoamine reuptake inhibitors, sex hormones, antipsychotics, and antihistamines, indicating an effect on MDD and potential repurposing opportunities. These findings not only require validation in model systems and clinical examination, but also show that GWAS may become a rich source of new therapeutic hypotheses for MDD and other psychiatric disorders that need new—and better—treatment options.

## Introduction

There is an urgent need for new drugs to better treat major depressive disorder (MDD), with new modes of action as well as fewer side effects. The Psychiatric Genomics Consortium (PGC) has conducted a genome-wide association study (GWAS) of more than 130,664 MDD and broader depression cases and 330,470 controls identifying 44 loci associated with depression^1^. Much new biology is suggested by these findings and we hypothesize that the collection of loci discovered by GWAS may have the potential to restart largely paused drug development pipelines. This is not without considerable technical challenges. At the moment, time-consuming manual assessment by expert biologists and geneticists is required for each GWAS locus. Analyzing all genome-wide results together may allow better prioritization of potential drug or therapeutic hypotheses^2,3^.

GWAS associations between single nucleotide polymorphisms (SNPs) and MDD can be used to assess the association of each gene or sets of genes, such as those defined by biological pathways. Pathway analysis has also been used to suggest new drug hypotheses by mapping drugs to the proteins they bind, and defining the sets of genes that encode the proteins as “drug gene-sets” whose association with a phenotype of interest can be estimated^2,4^. This process is a type of drug repositioning analysis aimed at finding potential new uses for existing drugs^2^. In this paper, we propose to mine drug-protein/gene interactions from two main sources: drug-target relationships or “activity profiles”^2^ and drug effects on gene expression or “perturbagen signatures”^5^. Activity profiles can be derived from several databases such as PubChem BioAssays^6^ or ChEMBL^7^, while the main source for perturbagen signatures is the CMAP database^5^. Instead of using these resources separately, they can be used together to identify relevant drugs. However, simply generating the association between drug-gene-sets and phenotypes is not enough; each gene-set is a subnetwork with different interaction types between drugs and proteins. Visualising these interactions could allow better and more rapid prioritization of drug-gene-sets.

For this purpose, it may be useful to translate activity profiles into bipartite drug-target interaction networks. These can be constructed by linking drug nodes to targets nodes where the links or edges represent the type of drug-target interaction. Maggiora et al.^8^ suggested that these networks could be used to assess drug polypharmacology—the ability of drugs to interact with several targets—as well as target polyspecificity—the ability of targets to exhibit affinity towards multiple dissimilar molecular compounds.

In this paper, we build drug-target networks relevant to a given phenotype (MDD), by using the results from a well-powered PGC MDD GWAS for imputation of tissue-specific expression levels in patients vs. controls, and to generate genetic associations of known drug targets with MDD. Drug-target networks presented in this paper can be accessed on the Drug Targetor website (drugtargetor.com), which provides the opportunity to build networks linking genetic data with a large number of drugs and drug classes, allowing detailed assessment of drug action possibly impacting MDD.

## Materials and methods

### Genome-wide association study of major depressive disorder

The PGC MDD phase 2 analysis was a combined analysis of an anchor cohort of traditionally ascertained MDD cases and an expanded cohort of more diversely assessed depression cases. Briefly, the anchor cohort consisted of 29 samples of European ancestry (16,823 MDD cases and 25,632 controls)^1^. Cases in the anchor cohort were required to meet international consensus criteria (DSM-IV, ICD-9, or ICD-10)^9^ for a lifetime diagnosis of MDD. Controls were screened for the absence of lifetime MDD (22/29 samples). An “expanded” set of six independent, European-ancestry cohorts (113,841 MDD cases and 304,838 controls) were then considered. Generation Scotland employed direct interviews; iPSYCH (Denmark) used national treatment registers; deCODE (Iceland) used national treatment registers and direct interviews; GERA used Kaiser-Permanente (health insurance) treatment records (CA, US); UK Biobank combined self-reported MDD symptoms and/or treatment for MDD by a medical professional; and 23andMe used self-report of treatment for MDD by a medical professional. All controls were screened for the absence of MDD. A combination of polygenic scoring and linkage disequilibrium (LD) score genetic correlation comparisons between the anchor and expanded cohorts and samples showed strong evidence for genetic homogeneity between these groups^1^.

### GWAS quality control and analysis

See ref. ^1^ for full details. SNPs and insertion-deletion polymorphisms were imputed using the 1000 Genomes Project multi-ancestry reference panel^10^. In each cohort, logistic regression association tests were conducted for imputed marker dosages with principal components covariates to control for population stratification. Ancestry was evaluated using principal components analysis applied to directly genotyped SNPs^11^. Summary statistic for 10,468,942 autosomal SNPs were then available for the analyses we present.

### Gene-based test of association

We used MAGMA v1.06^12^ to perform a gene-based test of association with the MDD GWAS summary statistics. Briefly, MAGMA generates gene-based *p*-values by combining adjacent SNP-based *p*-values within a defined gene window while accounting for LD. SNPs were mapped to genes if they were located 35 kb upstream or 10 kb downstream of a gene body including regulatory regions, and the gene *p*-value is obtained using the “multi = snp-wise” option, which aggregates mean and top SNP association models. A Bonferroni p-value threshold of 2.63 × 10^−6^, accounting for 19,079 ENSEMBL genes, was used to account for multiple testing. We used 1000 Genomes European data phase 3 as the reference LD set^10^.

### Transcriptome-wide association

To assess the impact of genetic variation underlying MDD on gene expression, we performed a transcriptome-wide association study using the S-PrediXcan software^13^. This approach estimates gene expression weights by training a linear prediction model in samples with both gene expression and SNP genotype data. The weights are then used to predict gene expression from GWAS summary statistics, while incorporating the variance and covariance of SNPs from a LD reference panel. We used pre-computed gene expression weights for 13 central nervous system (CNS) tissues (amygdala, anterior cingulate cortex BA24, caudate nucleus, nucleus accumbens, putamen, cerebellar hemisphere, cerebellum, cortex, frontal cortex BA9, hippocampus, hypothalamus, cervical spine C1, and substantia nigra) generated from the Genotype-Tissue Expression (GTEx) Consortium v7^14^, and whole blood using the Depression Genes and Networks (DGN) cohort^15^. Only CNS tissues were considered to investigate potential drug targets for MDD; however, the DGN whole blood analysis was also performed because of its higher sample size (922 whole blood samples) and higher power to detect associations. 1000 Genomes European data phase 3 was used as the reference LD set^10^. These data were processed with beta values and standard errors from the MDD GWAS summary statistics to estimate the expression-GWAS association statistic. A transcriptome-wide significance threshold of *P* *=* 1.12 × 10^−6^, adjusting for all GTEx CNS tissue and DGN associations (Bonferroni correction 0.05/44,718), was used to adjust for multiple testing.

### Definition of the druggable genome

Genes were annotated by “druggability” using the collection of 4479 “druggable genes” from Finan et al.^16^ (henceforth referred to as the “druggable genome”) and divided into three “tiers” based on their importance in pharmaceutical development: tier 1 (T1) contains genes that encode protein targets of approved or clinical trial-phase drug candidates, tier 2 (T2) contains genes that encode protein targets with high sequence similarity to tier 1 proteins or targeted by small drug-like molecules, and tier 3 contains genes that encode secreted and extracellular proteins, genes belonging to the main druggable gene families, and genes encoding proteins with more restricted similarity to tier 1 targets. In the gene-based tests of association, genes were investigated whether or not they were present in this druggable genome; results in Supplementary Materials are ordered by druggability tier. It should be noted that targets not known as “druggable” are also worth investigating—“druggability” is a mutable concept, which evolves as more drug-target data is made available. Information on genes with human or mouse phenotypes were also collected from the human-mouse disease connection database (HDMC), which gathers mouse data from Mouse Genome Informatics database (MGI)^17^ and human data from the National Center for Biotechnology Information (NCBI) and Online Mendelian Inheritance in Man (OMIM)^18^.

### Definition of drug-target and drug-gene interactions

We collected two types of drug interactions: activity profiles (drug-target interactions) and perturbagen signatures (drug-gene interactions). Drug-target interactions are defined as any type of interaction between a drug and a protein target. Drug-gene interactions are changes in gene expression induced by a drug. We built an annotation dataset using interaction profiles from the drug-gene interaction database DGIdb v2.0^19^, ChEMBL v.23^7,20^, the psychoactive drug-gene database PDSP *K*_i_ DB, PHAROS^21^, NCBI PubChem BioAssay^6^, and DSigDB^16,22^ (downloaded in June 2017). We subset experimental data from the annotation dataset to generate a more reliable bioactivity dataset, with only curated ChEMBL and PDSP *K*_i_ DB data. The broad annotation set was used to rank the drugs, but the bioactivity subset was used to check which drug classes were enriched when restricting analyses to curated experimental data. A description of the data curation approach is provided in Supplementary Text 1.

### Enrichment of drug-gene-sets and therapeutic classes

Approved drugs and their Anatomical Therapeutic Chemical (ATC) codes were identified by mapping all drug names to their PubChem compound identifier (CID) using the PubChem synonym database (ftp.ncbi.nlm.nih.gov/pubchem/Compound/Extras/CID-Synonym-filtered.gz), then mapping each CID to the corresponding ATC codes. The drugs were merged by ATC name, which could correspond to several CID entries and ATC codes. Each drug was then mapped to a gene-set using the collected drug-gene and drug-target interactions, and assigned a *p*-value generated by competitive pathway analysis (MAGMA), assessing the association between drug-gene-set and phenotype. For the annotation set, 1946 drugs corresponding to 1748 individual gene-sets were tested; for the more reliable bioactivity set of ChEMBL and PDSP *K*_i_ data, 1160 drugs were mapped to 723 gene-sets. The area under the enrichment curve (AUC) and associated *p*-value from one-sided Mann–Whitney–Wilcoxon (MWW) tests were used to assess the enrichment of drug classes, for each ATC hierarchical level. Since the annotation and bioactivity sets generated two different sets of pathway analysis *p*-values (drug ranks), MWW enrichment tests were carried out two times. The drugs were first ranked by MAGMA pathway analysis *p*-value, and the MWW test compared the drugs within one ATC level *A* to drugs not present in *A*, the alternative hypothesis being that the distribution of drugs in *A* is shifted to the right. The MWW test is adapted in this case since we have one continuous dependent variable (the pathway analysis *p*-value), and one independent variable consisting of two independent groups, with no overlap of instances between the two groups (drugs outside or inside a specific class). The Bonferroni threshold was estimated by dividing 0.05 by the number of tested classes—57 and 141 for the bioactivity and annotation sets, respectively.

### Bipartite drug-target networks

**Fig. 1:**
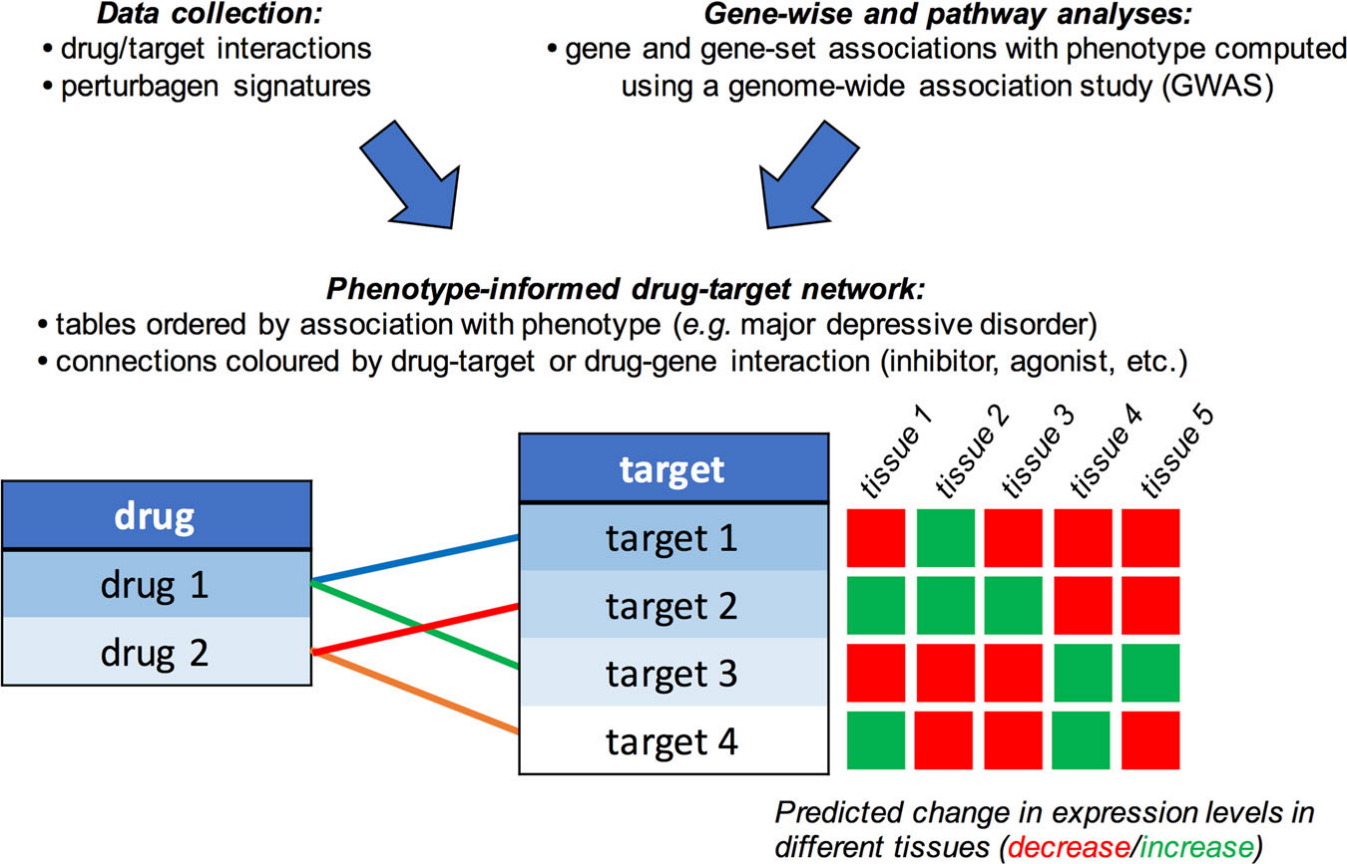
Drug Targetor workflow to build phenotype-informed bipartite drug-target networks (drugtargetor.com)

Bipartite drug-target networks were built using Drug Targetor (drugtargetor.com)^23^. The tool builds networks using three types of inputs: a drug table with drug-phenotype associations, a target table with target-phenotype associations, and connections between drugs and targets (cf. Figure 1). The drug-phenotype associations were obtained using MAGMA pathway analysis, target-phenotype associations using MAGMA gene-wise analysis and S-PrediXcan results, and the drug-target interactions were collected as described in the data collection section (cf. “Definition of drug-target and drug-gene interactions”). The networks are comprised of drug nodes and target nodes, the edges of which are connected based on the type of interaction. Drug Targetor defines nine types of drug-target interactions: increasing gene expression, decreasing gene expression, mixed (increasing or decreasing) gene expression, agonist/activator/positive allosteric modulator, partial agonist, antagonist/inhibitor/negative allosteric modulator, modulator (neither negative nor positive), inverse agonist, and mixed bioactivities (unknown or both agonist and antagonist). Only bioactivity data were used in the networks generated for this paper. The drug nodes are ordered by decreasing association with the phenotype in −log_10_(*P*) units (from MAGMA pathway analysis). The target nodes are ordered by a score based on MAGMA results and S-PrediXcan results in the tissue family of interest (for more details see https://drugtargetor.com/help.html). In Drug Targetor, the data entry for the new PGC MDD GWAS is encoded as “DEPR01”, and we chose the tissue family “Nervous System”.

## Results

### Gene-based tests of association

We used MAGMA to map SNP-level association to individual genes, and annotated results based on druggability (cf. “Definition of the druggable genome”). A total of 211 genes achieved genome-wide significance (MAGMA *P* ≤ 2.62 × 10^−6^, cf. Supplementary Tables 1–3), of which 153 were located outside the major histocompatibility complex (MHC) region. 24 of these 153 genes were annotated as druggable in the Finan et al. classification (Table 1). The MHC region was defined as the 25–35 Mb region on chromosome 6.

**Table 1:** “Druggable” genes outside the major histocompatibility complex significant in major depressive disorder. The −log_10_(*P*) column indicates the significance level as computed by MAGMA, the DGN whole blood and GTEx brain regions columns indicate the predicted change in expression level in the corresponding tissue

Gene name	−log_10_(*P*)	DGN whole blood	GTEx bain regions
*NEGR1*	16.07	+^a^	+, +^a^
*OLFM4*	15.54	+	+, +
*CACNA1E*	10.23	−	
*PXDNL*	8.92	−	+
*ESR2*	8.70	−^a^	−
*EP300*	8.15	+	
*VRK2*	8.10	+	−
*DRD2*	8.04		
*CACNA2D1*	8.03		+, −
*CHALD*	7.96	+	+
*ENOX1*	7.70		+, −
*EMILIN3*	7.27		−, −, −
*HP*	7.08	−	+, +
*GRM5*	6.71		+, +
*FEN1*	6.66	−	
*PCDHA2*	6.62		+, −
*RPS6KL1*	6.60	+	−
*KCNB1*	6.56		
*PCSK5*	6.34	+	−
*HSPD1*	6.14	−	
*GRIK5*	6.04	−	
*WDR1*	5.94	+	−, −
*HTR1D*	5.70		+, +, +, +, +, +
*LINGO1*	5.69		+, +, +,−, −, −

+predicted upregulation in one brain region, −predicted downregulation in one brain region

^a^Bonferroni-significant

To gain insight into the potential functional consequences of DNA sequence variation underlying MDD, we imputed gene expression using S-PrediXcan. Overall, 12 protein-coding genes outside the MHC were significantly up or downregulated in the brain or whole blood, (Supplementary Table 4). Among these genes, five were significant in the brain: *NEGR1*, *LRFN5*, *KCL1*, *TMEM33* (upregulation), and *SLC30A9* (downregulation), and three were annotated as druggable: *NEGR1*, *LRFN5*, and *ESR2*. *NEGR1* is positively associated with MDD in the putamen (*Z* = 7.06, *P* = 1.67 × 10^−12^), *LRFN5* in the cerebellum (*Z* = 5.21, *P* = 2.01 × 10^−11^) and cerebellar hemisphere (*Z* = 5.23, *P* = 1.68 × 10^−7^), whereas *ESR2* is not significantly associated with MDD in the brain (cerebellum: Z = −1.73, *P* = 0.0843) but exhibits significant negative association in whole blood (*Z* = −5.43, *P* = 5.66 × 10^−8^).

### Drug classes and their drug-target networks

**Fig. 2:**
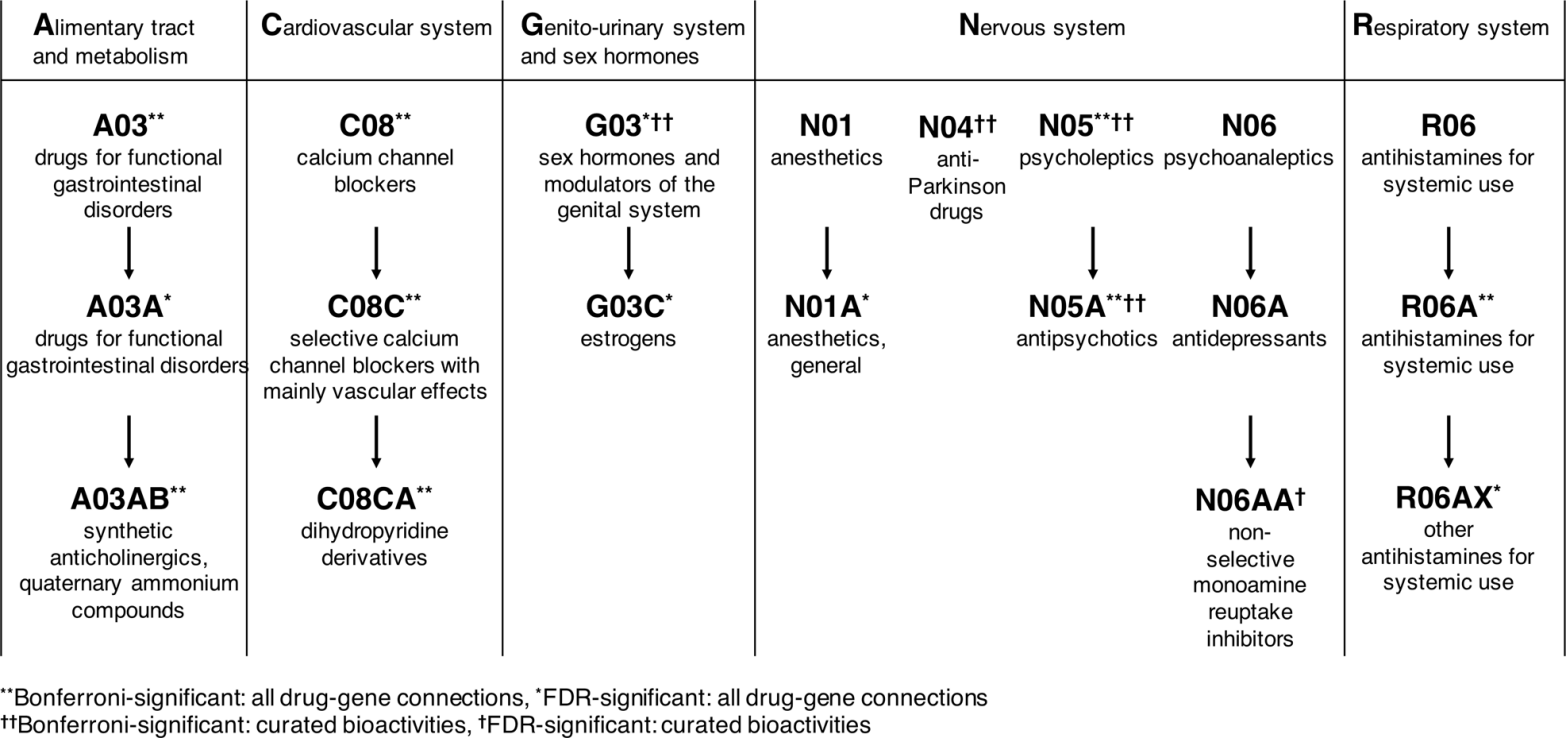
Drug classes significantly enriched (FDR or Bonferroni) in major depressive disorder when using the complete annotation set (“all drug-gene connections”), or using only the curated bioactivity set (“curated bioactivities”), which contains ChEMBL and PDSP Ki data. The drug class enrichment was tested using Mann–Whitney–Wilcoxon (MWW) rank tests, on drugs ordered by MAGMA pathway analysis *p*-value. The Bonferroni correction is based on the number of tested classes: 57 for the curated bioactivity set, 141 for the complete connection set

We tested for the enrichment of MDD GWAS association signals within major therapeutic classes defined by ATC code, using the complete set of drug-gene interactions (“annotation” dataset) or only curated ChEMBL and PDSP bioactivities (“bioactivity” dataset). We used the Benjamini and Hochberg false discovery rate (FDR)^24^ to correct for multiple testing whilst exploring more hypotheses (Fig. 2 and Supplementary Table 5). In the annotation dataset, 13 drug classes were significantly associated with MDD (FDR *q*-value < 0.05); in the more reliable bioactivity dataset, only five classes were identified as significant (Fig. 2**)**. Both annotation and bioactivity datasets exhibited enrichment for psycholeptics (ATC code N05, *P*_bioactivity_ = 9.81 × 10^−6^), antipsychotics (N05A, *P*_bioactivity_ = 1.71 × 10^−5^), and sex hormones and modulators of the genital system (G03, *P*_bioactivity_ = 5.75 × 10^−5^). Non-selective monoamine reuptake inhibitors (N06AA, a subclass of antidepressants) were only significant in the bioactivity dataset (*P*_bioactivity_ = 3.23 × 10^−3^).

Bipartite drug-target networks, which provide an insight into the mode of action for drugs and their putative targets, were built for each FDR-significant drug class (Supplementary Figures 1–15). These networks provide a visual support for the drug class enrichment analysis, by highlighting the drug-target interactions driving the association between drugs in an enriched class and MDD—they also suggest in which tissues the genes might be up or downregulated and the potential negative or positive action of a drug. Phenotypic information from the human-mouse disease connection (HDMC) database for prioritized targets was also collected as supplementary information and provided in Table 2. Four patterns occur most often among Bonferroni-significant drug classes: dopamine receptor D2 antagonism/agonism (*DRD2*), serotonin receptor 5-HT1D antagonism/agonism (*HTR1D*), calcium channels (*CACNA2D1* and *CACNA1H*, *CACNA1C* being only FDR-significant) modulation and antagonism, and estrogen receptor ER-β (*ESR2*) modulation. Other patterns seen for FDR-significant drug classes include: cholinergic/acetylcholine receptor M3 antagonism (*CHRM3*), estrogen receptor ER-α (*ESR1*) modulation, GABA-A receptor agonism and antagonism (subunits encoded by *GABRA1*, *GABRG3*, *GABRA6*), histamine H1 receptor antagonism (*HRH1*), and glutamate receptor 1 antagonism (*GRIA1*). A detailed description of druggable targets and their interactions is provided in Supplementary Text 2.

**Table 2:** Hub targets in drug-target networks, with human (H) or mouse (M) phenotypes identified in the HDMC (human-mouse disease connection database)

Gene	Target	Significance	Behavior, neurological	Nervous system	Drug classes
*DRD2*	Dopamine receptor D2	***	H/M	H/M	Drugs for gastrointestinal disorders, psycholeptics, antipsychotics, psychoanaleptics
*HTR1D*	Serotonin receptor 5-HT1D	**	Normal mouse phenotype	Normal mouse phenotype	Antipsychotics, analgesics (triptans)
*CACNA2D1*	Calcium channel subunit	***	M	M	Calcium channel blockers/modulators
*CACNA1H*	Calcium channel subunit	**	M	H/M	Calcium channel blockers/modulators
*ESR2*	Estrogen receptor ER-β	***	H/M	H/M	Hormones and modulators of the genital system
*CHRM3*	Cholinergic/acetylcholine receptor M3	*	M	H/M	Drugs for gastrointestinal disorders, antipsychotics, psychoanaleptics
*ESR1*	Estrogen receptor ER-α	*	H/M	H/M	Hormones and modulators of the genital system
*GABRA1*	GABA-A receptor subunit	*	M	H/M	Anesthetics, psycholeptics
*GABRG3*	GABA-A receptor subunit	*	–	–	Anesthetics, psycholeptics
*GABRA6*	GABA-A receptor subunit	*	M	M	Anesthetics, psycholeptics
*HRH1*	Histamine H1 receptor	*	M	–	Antihistamines, antipsychotics, psychoanaleptics
*GRIA1*	Glutamate receptor 1	*	M	M	Anesthetics
*CACNA1C*	Calcium channel subunit	*	M	H/M	Calcium channel blockers/modulators

*H* human, *M* mouse

***Bonferroni-significant MAGMA results (−log_10_(*p*) > 5.58), **Bonferroni-suggestive (−log_10_(*p*) > 4.58), *FDR-sgnificant (*q*-value < 0.05)

### Potential repurposing candidates

**Fig. 3:**
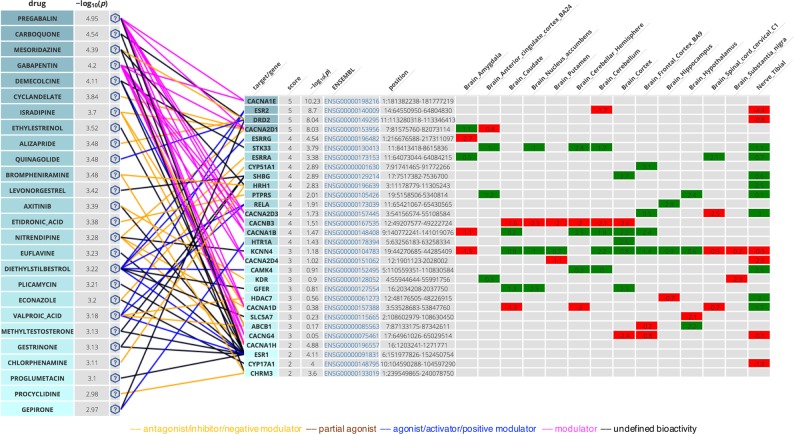
Drug-target network from the online tool Drug Targetor (drugtargetor.com), showing the top drugs and their top-classified targets/genes for MDD in the Nervous System, ordered by genetic scores. The drug association statistics in −log_10_(*P*) units were obtained using MAGMA pathway analysis, and the gene associations using MAGMA gene-wise analysis. Expression z-scores obtained by S-PrediXcan in the different tissues are colored in green for positive effect, red for negative effect. Drug/target connections are colored by drug action type

Top individual drugs from pathway analyses that have interaction with significant targets (Fig. 3) include calcium channel modulators or blockers (such as pregabalin, gabapentin, and nitrendipine), dopamine receptor D2 antagonists (alizapride, mesoridazine) or agonists (quinagolide), and hormonal medications such as levonorgestrel (inhibitory effect on sex hormone binding globulin) or diethylstilbestrol, an agonist of estrogen receptors. Gepirone is the only antidepressant in the top list and its association with MDD is driven by its dopamine D2 binding and 5-HT1A partial agonism. Other potentially more interesting candidates can be found by visualizing each enriched drug class in a bipartite drug-target interaction network (cf. Supplementary Figures 1–15 and Discussion).

## Discussion

Most antidepressants are only partially effective and not all patients respond to these treatments, which also have frequent side effects that contribute to reduced treatment adherence^25^. Therefore, the antidepressant suitable for the individual patient is mostly chosen based on its efficacy and side effect profile in a strenuous and time-consuming process. Using the largest available GWAS, we conducted systematic analyses for associations of MDD with known drug targets and drug classes. We find that 13 drug classes based on the ATC classification are enriched for associations in the MDD GWAS data, among which are antidepressants, antipsychotics as well as sex hormones and antihistamines. We visualise and explore these drug classes using our online tool Drug Targetor (drugtargetor.com), which displays bipartite drug-target networks for MDD that integrate genetic association and imputed gene expression information. The imputed gene expression data may indicate up or downregulation of genes in different tissues, thereby suggesting whether the effect of a gene or target should be inhibited or enhanced—for example, an antagonist could counteract the effect of an overexpressed target in the brain.

We identified association patterns for MDD concentrated around key drug-target hubs, including calcium channels, dopamine, serotonin, histamine, and GABA receptors, as well as the predominantly female sex hormone estrogen. Many of the top druggable genes encode subunits of voltage-dependent calcium channels expressed in the brain (*CACNA2D1*, *CACNA1H*, *CACNA1C*), or are receptors of neurotransmitters and their subunits, such as GABA (*GABRA1*, *GABRG3*, *GABRA6*), acetylcholine (*CHRM3*), glutamate (*GRIA1*, *GRM5*, *GRM8*, *GRIK5*), serotonin (*HTR1D*), and dopamine (*DRD2*). These neurotransmitter receptors are targeted by many drugs included in the psycholeptics, psychoanaleptics, and anesthetics drug classes, many of which are already approved for the treatment of MDD.

The enrichment of calcium channels confirms that calcium channel blockers such as verapamil may provide repurposing opportunities for MDD^26^, although their effects on blood pressure may prove problematic^27^. Pregabalin and gabapentin, both calcium channel modulators, are also top ranked repurposing candidates. Pregabalin has been shown to be an effective adjunctive treatment for MDD^28^ and treatment-resistant bipolar disorder^29^, and gabapentin is used off-label for bipolar disorder^30^. The side effect profile of gabapentin includes increased suicidality within the first week of treatment^31^, which is also seen with antidepressant use. The mood elevating effect of antidepressants is thought to occur after about 2–3 weeks, lagging the increase in motivational behaviour, which could explain the higher risk for suicidal attempts^32,33^. It may be that administration of calcium channel modulators over a longer time period could lead to a decrease of depressive symptoms after overcoming an initial ineffective episode.

The association of histamine receptor H1 with MDD may indicate an involvement of the histaminergic system in MDD and depressive symptoms. Brompheniramine and chlorphenamine, which have very similar structures, are the top antihistamines associated with MDD. Interestingly, brompheniramine is the precursor of one of the first marketed antidepressant compounds, zimelidine, the first selective serotonin reuptake inhibitor (SSRI), patented in 1972^34^, although no longer in use due to its side effect profile^35^. These medications are first-generation antihistamines, which exhibit sedating effects of different intensities, which may help with disrupted sleep, a symptom common in MDD patients^36^.

A female preponderance in MDD is well-established^37,38^, making sex hormones interesting candidates for the treatment of MD. We saw associations between the estrogen receptors (ERs) *ESR1* and *ESR2* and MDD. This finding was further supported by a significant association of decreased whole-blood *ESR2* expression and MDD, indicating that ER-β agonism could be possibly beneficial. However, no significant associations with altered expression levels in brain regions were found—which could be due to a lack of power. Lasofoxifene was a top ranked selective estrogen receptor modulator (SERM) identified in our drug-target networks. SERMs are hypothesised to function as neuroprotective and antiinflammatory agents in the central nervous system^39^ and the SERM raloxifene has been reported to decrease anxiety^40^ and depression^41^. Among sex hormones, levonorgestrel is one of our top repurposing candidates. The use of a levonorgestrel in intrauterine systems was associated with lower risk of postpartum depression^42^; however, another study showed increased risk of antidepressant use and first diagnosis of MDD^43^.

Ketamine, a member of the drug class of anesthetics, is used off-label for depression via intravenous infusions^44,45^; our results suggest that its D2 partial agonism might be one possible explanation for its antidepressant effect, together with its serotonin and glutamate receptor antagonism^46^ and interaction with other neurotransmitter systems^47^. In our analyses of druggable genes, the dopamine receptor 2 (D2) gene (*DRD2*) is clearly associated with MDD. In addition, antipsychotics as well as antidepressants targeting D2 are usually antagonists; antipsychotics are used as augmentation therapies in patients with MDD if initial antidepressant therapies do not result in remission of symptoms^48^. However, we note that mesoridazine, a neuroleptic and D2 antagonist in our top list for repurposing opportunities, was withdrawn from the US market due to major side effects^49^.

Our analyses suggest that *HTR1D* overexpression is associated with MDD in six brain regions (although none of these associations is significant). *HTR1D* overexpression could either be leading to depressive symptoms, suggesting that 5-HT1D antagonism could counteract them, or could be a compensatory mechanism due to low serotonin levels, suggesting a beneficial effect of 5-HT1D agonists on depressive symptoms. The first hypothesis is supported by the 5-HT1D antagonist activity displayed by vortioxetine, an antidepressant and serotonin modulator^50^.

These results, while interesting, have considerable caveats. Specifically, a key point when using GWAS data is the direction of effect. The relationship between a drug and a phenotype cannot easily be inferred; an association may reflect either a depression-inducing effect or an antidepressant effect. We partially address this issue via imputation and prediction of gene expression, but pharmacological, molecular and clinical validation will be needed before drawing definitive conclusions. However, we suggest that our findings may represent a source of new therapeutic hypotheses for MDD—a common and currently only partially treatable disorder.

## Supplementary information

Supplementary Texts 1-3.

Supplementary Tables 1-6.

Supplementary Figures 1-15.
