# Supplementary material for: Using genetic drug-target networks to develop new drug hypotheses for major depressive disorder
Source: Transl Psychiatry. 2019 Mar 15;9:117. doi: 10.1038/s41398-019-0451-4 (PMC6420656; doi:10.1038/s41398-019-0451-4)
Supplement: Supplementary file 1 — Supplementary Texts 1-3. [file 41398_2019_451_MOESM1_ESM.pdf]

Using genetic drug-target networks to develop new drug hypotheses for major depressive disorder: Supplement 1

Héléna A Gaspar PhD, Zachary Gerring PhD, Christopher Hübel MD MSc, Major Depressive Disorder Working Group of the Psychiatric Genomics Consortium, Christel M Middeldorp MD PhD, Eske M Derks PhD, Gerome Breen PhD

Supplementary Text 1: Data curation: bioactivities

Drug-target bioactivities were mined from the following databases: PDSP Ki DB downloaded in June 2017 [1], PHAROS[2] (https://pharos.nih.gov/download/tcrd_v3.0.7.sql.gz), NCBI PubChem BioAssay[3] processed by ChemMine Tools (http://biocluster.ucr.edu/~tbackman/bioassayR/pubchem_protein_only.sqlite), and ChEMBL v.23[4,5].

In PHAROS, ChEMBL and K_i DB only EC50, IC50, AC50, Potency, K_i, and K_d affinity types were collected and independently curated. We converted each activity type A to pA = $-\log_{10}(A)$ and curated them separately to generate unique drug-target pairs within each database, and then merged the different datasets. For each activity type in each separate resource, we only kept drug-target interactions satisfying both conditions:

1. Activity threshold: $6 \leq pA < 14$
2. Activity error: $\Delta pA < 2$, $\Delta pA = pA(max) - pA(min)$

Where ΔpA is the range of pA activities, *i.e.*, the difference between max and min pA values for each drug-target pair. This means that the activity of a drug against a target should be at least 1 μM and not smaller than 10^{-5} nM to avoid covalent binding. In PubChem, data was filtered based on the PubChem activity score (> 50), using the BioAssayR database from ChemMine tools[6], updated April 6th 2016.

Supplementary Text 2: Main interactions between druggable targets and drug classes

A) Dopamine receptor D2 antagonism/agonism

A03 drugs (gastrointestinal disorders) bromopride, domperidone or metoclopramide are D2 protein product antagonists. The anesthetic drug ketamine is a partial D2 agonist. Psycholeptics and psychoanaleptics both show a concentration of D2 interactions; however, antipsychotic drugs are mostly D2 antagonists, whereas psychoanaleptics can be agonists (minaprine, memantine, dexamfetamine) or antagonists (amoxapine, doxepin, nortriptyline, nomifensine, mianserin).

B) Serotonin receptor 5-HT1D antagonism/agonism

HTR1D is predicted to have an increased expression profile across brain regions - however, it is not significant. *HTR1D* encodes 5-hydroxytryptamine (serotonin) receptor 1D (5-HT1D) and is targeted by several drug classes. Vortioxetine (an antidepressant) is a known antagonist and other antidepressants (mianserin and trazodone) have a recorded affinity for that target. Several antipsychotics are also known 5-HT1D antagonists. On the other hand, several analgesics (triptans) are 5-HT1D agonists, such as ergotamine (also a D2 agonist). Aripiprazole (antipsychotic), olanzapine (antipsychotic) and vortioxetine (antidepressant) are 5-HT1D antagonists. On the other hand, many analgesic drugs are 5-HT1D agonists and could have an impact on MDD: ergotamine (also a D2 agonist), naratriptan, sumatriptan, rizatriptan, eletriptan, almotriptan, frovatriptan, zolmitriptan, and dihydroergotamine.

C) Calcium channel modulation and antagonism

CACNA1H encodes the pore-forming subunit (α1) Cav3.2, a voltage-gated T-type calcium channel (low voltage activated), and *CACNA2D1* encodes a member of the α2δ subunit family and is differentially expressed in the brain[7]. Calcium channel blockers might have a positive effect on MDD through *CACNA2D1* and *CACNA1H*: *CACNA1H* over-expression in the caudate, nucleus accumbens and cerebellum might be associated with MDD. C08 drugs (calcium channel blockers) are calcium channel modulators and antagonists (top genes *CACNA2D1*, *CACNA1H* and *CACNA1C*). Top drugs include nitrendipine, isradipine and nicardipine.

D) ER-α and ER-β modulation and agonism

Several G03 drugs (sex hormones and modulators of the genital system) are ER-β and ER-α modulators. Progesterone might also upregulate ER-β.

E) Muscarinic receptor M3 and histamine receptor H1 antagonism

Several drugs for gastrointestinal disorders, as well as antipsychotics and psychoanaleptics are M3 antagonists. Several antipsychotics, psychoanaleptics as well as antihistamines are H1 antagonists.

F) GABA-A receptors agonism/antagonism (GABRA1, GABRG3, GABRA6) and glutamate ionotropic receptor AMPA type (GRIA1) antagonism

The association between anesthetics and MDD is driven by GABA-A receptor agonism (*GABRA1*, *GABRG3*, *GABRA6*) and glutamate receptor antagonism (*GRIA1*). Psycholeptics are also GABA-A receptor agonists (top genes *GABRA1, GABRG3*).

G) Isolated connections, taking into account drug perturbagen signatures

Amongst antidepressant drugs, only amitriptyline is listed as BDNF/NT-3 growth factors receptor (*NTRK2*) agonist. Amongst G03 drugs (hormones and modulators of the genital system), danazol downregulates *VRK2* (also associated with schizophrenia) and upregulates *GRIK5*. Paracetamol (analgesic) is not listed as a 5-HT1D agonist but might downregulate the expression of several MDD-related genes such as *GRM5*, *KMO*, *DHODH*, and upregulate *FEN1*. Zuclopenthixol (psycholeptic) upregulates *GRM8*. Progesterone and estradiol might be downregulators of *NEGR1*.

Supplementary Text 3: Note on ***VRK2, LINGO1, GRM5, GRM8 and GRIK5***

GRM5, *GRM8* and *GRIK5* are involved in glutamatergic neurotransmission. *GRIK5* encodes glutamate receptor ionotropic kainate 5. *GRM5* and *GRM8* encode glutamate metabotropic receptors 5 and 8. A human PET study (Positron Emission Tomography) suggested reduced GRM5 neurotransmission in depression; a post-mortem study showed reduced expression in the

frontal cortex of depressed patients[8]. However, these studies have very small sample sizes - 10 cases for the PET study and 15 cases for the post-mortem study. Mouse models indicate that *GRM5* suppression might help to normalize social interactions[9].

VRK2 encodes a member of the vaccinia-related serine/threonine-protein kinase, and its role in mood disorders remains unclear. However, *VRK2* expression has recently been investigated by Tesli et al:[10] mRNA levels were found to be lower in schizophrenia and bipolar disorders. *LINGO1* is an interesting target. It shows significant association with neuroticism, and our results show a mixed predicted expression pattern in the brain (all non-significant). A study by Fernandez-Enright et al.[11] has demonstrated altered *LINGO1* signalling in the brain and suggested the use of *LINGO1* antagonists for schizophrenia therapies.

References

1 Roth BL, Lopez E, Patel S, Kroeze WK. The Multiplicity of Serotonin Receptors: Uselessly Diverse Molecules or an Embarrassment of Riches? *Neuroscientist* 2000; **6**: 252–262.

2 Nguyen D-T, Mathias S, Bologa C, Brunak S, Fernandez N, Gaulton A *et al.* Pharos: Collating protein information to shed light on the druggable genome. *Nucleic Acids Res* 2017; **45**: D995–D1002.

3 Wang Y, Cheng T, Bryant SH. PubChem BioAssay: A Decade's Development toward Open High-Throughput Screening Data Sharing. *SLAS DISCOVERY: Advancing Life Sciences R&D* 2017; **22**: 655–666.

4 Bento AP, Gaulton A, Hersey A, Bellis LJ, Chambers J, Davies M *et al.* The ChEMBL bioactivity database: an update. *Nucleic Acids Res* 2014; **42**: D1083–90.

5 CHEMBL database release 23. 2017 doi:10.6019/chembl.database.23.

6 Backman TWH, Cao Y, Girke T. ChemMine tools: an online service for analyzing and clustering small molecules. *Nucleic Acids Res* 2011; **39**: W486–91.

7 Dolphin AC. The α2δ subunits of voltage-gated calcium channels. *Biochim Biophys Acta* 2013; **1828**: 1541–1549.

8 Terbeck S, Akkus F, Chesterman LP, Hasler G. The role of metabotropic glutamate receptor 5 in the pathogenesis of mood disorders and addiction: combining preclinical evidence with human Positron Emission Tomography (PET) studies. *Front Neurosci* 2015; **9**: 86.

9 Chung W, Choi SY, Lee E, Park H, Kang J, Park H *et al.* Social deficits in IRSp53 mutant mice improved by NMDAR and mGluR5 suppression. *Nat Neurosci* 2015; **18**: 435–443.

10 Tesli M, Wirgenes KV, Hughes T, Bettella F, Athanasiu L, Hoseth ES *et al.* VRK2 gene expression in schizophrenia, bipolar disorder and healthy controls. *Br J Psychiatry* 2016; **209**: 114–120.

11 Fernandez-Enright F, Andrews JL, Newell KA, Pantelis C, Huang XF. Novel implications of Lingo-1 and its signaling partners in schizophrenia. *Transl Psychiatry* 2014; **4**: e348.

Major Depressive Disorder Working Group of the Psychiatric Genomics Consortium (PGC MDD Group)

Naomi R Wray [1,2 †] , Stephan Ripke [3,4,5 †] , Manuel Mattheisen [6,7,8,9 †] , Maciej Trzaskowski [1 †] , Enda M Byrne [1] , Abdel Abdellaoui [10] , Mark J Adams [11] , Esben Agerbo [8,12,13] , Tracy M Air [14] , Till F M Andlauer [15,16] , Silviu-Alin Bacanu [17] , Marie Bækvad-Hansen [8,18] , Aartjan T F Beekman [19] , Tim B Bigdeli [17,20] , Elisabeth B Binder [15,21] , Douglas H R Blackwood [11] , Julien Bryois [22] , Henriette N Buttenschøn [7,8,23] , Jonas Bybjerg Grauholm [8,18] , Na Cai [24,25] , Enrique Castelao [26] , Jane Hvarregaard Christensen [6,7,8] , Toni-Kim Clarke [11] , Jonathan R I Coleman [27] , Lucía Colodro-Conde [28] , Baptiste Couvy-Duchesne [29,30] , Nick Craddock [31] , Gregory E Crawford [32,33] , Cheynna A Crowley [34] , Hassan S Dashti [3,35] , Gail Davies [36] , Ian J Deary [36] , Franziska Degenhardt [37,38] , Eske M Derks [28] , Nese Direk [39,40] , Conor V Dolan [10] , Erin C Dunn [41,42,43] , Thalia C Eley [27] , Nicholas Eriksson [44] , Valentina Escott-Price [45] , Farnush Farhadi Hassan Kiadeh [46] , Hilary K Finucane [47,48] , Andreas J Forstner [37,38,49,50] , Josef Frank [51] , Héléna A Gaspar [27] , Michael Gill [52] , Paola Giusti-Rodríguez [53] , Fernando S Goes [54] , Scott D Gordon [55] , Jakob Grove [6,7,8,56] , Lynsey S Hall [11,57] , Christine Søholm Hansen [8,18] , Thomas F Hansen [58,59,60] , Stefan Herms [37,38,50] , Ian B Hickie [61] , Per Hoffmann [37,38,50] , Georg Homuth [62] , Carsten Horn [63] , Jouke-Jan Hottenga [10] , David M Hougaard [8,18] , Ming Hu [64] , Craig L Hyde [65] , Marcus Ising [66] , Rick Jansen [19,19] , Fulai Jin [67,68] , Eric Jorgenson [69] , James A Knowles [70] , Isaac S Kohane [71,72,73] , Julia Kraft [5] , Warren W. Kretzschmar [74] , Jesper Krogh [75] , Zoltán Kutalik [76,77] , Jacqueline M Lane [3,35,78] , Yihan Li [74] , Yun Li [34,53] , Penelope A Lind [28] , Xiaoxiao Liu [68] , Leina Lu [68] , Donald J MacIntyre [79,80] , Dean F MacKinnon [54] , Robert M Maier [2] , Wolfgang Maier [81] , Jonathan Marchini [82] , Hamdi Mbarek [10] , Patrick McGrath [83] , Peter McGuffin [27] , Sarah E Medland [28] , Divya Mehta [2,84] , Christel M Middeldorp [10,85,86] , Evelin Mihailov [87] , Yuri Milaneschi [19,19] , Lili Milani [87] , Francis M Mondimore [54] , Grant W Montgomery [1] , Sara Mostafavi [88,89] , Niamh Mullins [27] , Matthias Nauck [90,91] , Bernard Ng [89] , Michel G Nivard [10] , Dale R Nyholt [92] , Paul F O'Reilly [27] , Hogni Oskarsson [93] , Michael J Owen [94] , Jodie N Painter [28] , Carsten Bøcker Pedersen [8,12,13] , Marianne Giørtz Pedersen [8,12,13] , Roseann E. Peterson [17,95] , Erik Pettersson [22] , Wouter J Peyrot [19] , Giorgio Pistis [26] , Danielle Posthuma [96,97] , Shaun M Purcell [98] , Jorge A Quiroz [99] , Per Qvist [6,7,8] , John P Rice [100] , Brien P. Riley [17] , Margarita Rivera [27,101] , Saira Saeed Mirza [40] , Richa Saxena [3,35,78] , Robert Schoevers [102] , Eva C Schulte [103,104] , Ling Shen [69] , Jianxin

Shi [105], Stanley I Shyn [106], Engilbert Sigurdsson [107], Grant C B Sinnamon [108], Johannes H Smit [19], Daniel J Smith [109], Hreinn Stefansson [110], Stacy Steinberg [110], Craig A Stockmeier [111], Fabian Streit [51], Jana Strohmaier [51], Katherine E Tansey [112], Henning Teismann [113], Alexander Teumer [114], Wesley Thompson [8,59,115,116], Pippa A Thomson [117], Thorgeir E Thorgeirsson [110], Chao Tian [44], Matthew Traylor [118], Jens Treutlein [51], Vassily Trubetskoy [5], André G Uitterlinden [119], Daniel Umbricht [120], Sandra Van der Auwera [121], Albert M van Hemert [122], Alexander Viktorin [22], Peter M Visscher [1,2], Yunpeng Wang [8,59,115], Bradley T. Webb [123], Shantel Marie Weinsheimer [8,59], Jürgen Wellmann [113], Gonneke Willemsen [10], Stephanie H Witt [51], Yang Wu [1], Hualin S Xi [124], Jian Yang [2,125], Futao Zhang [1], eQTLGen Consortium [126], 23andMe Research Team [44], Volker Arolt [127], Bernhard T Baune [14], Klaus Berger [113], Dorret I Boomsma [10], Sven Cichon [37,50,128,129], Udo Dannlowski [127], EJC de Geus [10,130], J Raymond DePaulo [54], Enrico Domenici [131], Katharina Domschke [132], Tõnu Esko [3,87], Hans J Grabe [121], Steven P Hamilton [133], Caroline Hayward [134], Andrew C Heath [100], David A Hinds [44], Kenneth S Kendler [17], Stefan Kloiber [66,135,136], Glyn Lewis [137], Qingqin S Li [138], Susanne Lucae [66], Pamela AF Madden [100], Patrik K Magnusson [22], Nicholas G Martin [55], Andrew M McIntosh [11,36], Andres Metspalu [87,139], Ole Mors [8,140], Preben Bo Mortensen [7,8,12,13], Bertram Müller-Myhsok [15,16,141], Merete Nordentoft [8,142], Markus M Nöthen [37,38], Michael C O'Donovan [94], Sara A Paciga [143], Nancy L Pedersen [22], Brenda WJH Penninx [19], Roy H Perlis [42,144], David J Porteous [117], James B Potash [145], Martin Preisig [26], Marcella Rietschel [51], Catherine Schaefer [69], Thomas G Schulze [51,104,146,147,148], Jordan W Smoller [41,42,43], Kari Stefansson [110,149], Henning Tiemeier [40,150,151], Rudolf Uher [152], Henry Völzke [114], Myrna M Weissman [83,153], Thomas Werge [8,59,154], Ashley R Winslow [155,156], Cathryn M Lewis [27,157 *], Douglas F Levinson [158 *], Gerome Breen [27,159 *], Anders D Børglum [6,7,8 *], Patrick F Sullivan [22,53,160 *], for the Major Depressive Disorder Working Group of the Psychiatric Genomics Consortium.

† Equal contributions. * Co-last authors.

1, Institute for Molecular Bioscience, The University of Queensland, Brisbane, QLD, AU

2, Queensland Brain Institute, The University of Queensland, Brisbane, QLD, AU

3, Medical and Population Genetics, Broad Institute, Cambridge, MA, US

4, Analytic and Translational Genetics Unit, Massachusetts General Hospital, Boston, MA, US

5, Department of Psychiatry and Psychotherapy, Universitätsmedizin Berlin Campus Charité Mitte, Berlin, DE
6, Department of Biomedicine, Aarhus University, Aarhus, DK
7, iSEQ, Centre for Integrative Sequencing, Aarhus University, Aarhus, DK
8, iPSYCH, The Lundbeck Foundation Initiative for Integrative Psychiatric Research,, DK
9, Centre for Psychiatry Research, Department of Clinical Neuroscience, Karolinska Institutet, Stockholm, SE
10, Dept of Biological Psychology & EMGO+ Institute for Health and Care Research, Vrije Universiteit Amsterdam, Amsterdam, NL
11, Division of Psychiatry, University of Edinburgh, Edinburgh, GB
12, Centre for Integrated Register-based Research, Aarhus University, Aarhus, DK
13, National Centre for Register-Based Research, Aarhus University, Aarhus, DK
14, Discipline of Psychiatry, University of Adelaide, Adelaide, SA, AU
15, Department of Translational Research in Psychiatry, Max Planck Institute of Psychiatry, Munich, DE
16, Munich Cluster for Systems Neurology (SyNergy), Munich, DE
17, Department of Psychiatry, Virginia Commonwealth University, Richmond, VA, US
18, Center for Neonatal Screening, Department for Congenital Disorders, Statens Serum Institut, Copenhagen, DK
19, Department of Psychiatry, Vrije Universiteit Medical Center and GGZ inGeest, Amsterdam, NL
20, Virginia Institute for Psychiatric and Behavior Genetics, Richmond, VA, US
21, Department of Psychiatry and Behavioral Sciences, Emory University School of Medicine, Atlanta, GA, US
22, Department of Medical Epidemiology and Biostatistics, Karolinska Institutet, Stockholm, SE
23, Department of Clinical Medicine, Translational Neuropsychiatry Unit, Aarhus University, Aarhus, DK
24, Statistical genomics and systems genetics, European Bioinformatics Institute (EMBL-EBI), Cambridge, GB
25, Human Genetics, Wellcome Trust Sanger Institute, Cambridge, GB
26, Department of Psychiatry, University Hospital of Lausanne, Prilly, Vaud, CH

27, MRC Social Genetic and Developmental Psychiatry Centre, King's College London, London, GB
28, Genetics and Computational Biology, QIMR Berghofer Medical Research Institute, Herston, QLD, AU
29, Centre for Advanced Imaging, The University of Queensland, Saint Lucia, QLD, AU
30, Queensland Brain Institute, The University of Queensland, Saint Lucia, QLD, AU
31, Psychological Medicine, Cardiff University, Cardiff, GB
32, Center for Genomic and Computational Biology, Duke University, Durham, NC, US
33, Department of Pediatrics, Division of Medical Genetics, Duke University, Durham, NC, US
34, Biostatistics, University of North Carolina at Chapel Hill, Chapel Hill, NC, US
35, Center for Genomic Medicine, Massachusetts General Hospital, Boston, MA, USA
36, Centre for Cognitive Ageing and Cognitive Epidemiology, University of Edinburgh, Edinburgh, GB
37, Institute of Human Genetics, University of Bonn, Bonn, DE
38, Life&Brain Center, Department of Genomics, University of Bonn, Bonn, DE
39, Psychiatry, Dokuz Eylul University School Of Medicine, Izmir, TR
40, Epidemiology, Erasmus MC, Rotterdam, Zuid-Holland, NL
41, Stanley Center for Psychiatric Research, Broad Institute, Cambridge, MA, US
42, Department of Psychiatry, Massachusetts General Hospital, Boston, MA, US
43, Psychiatric and Neurodevelopmental Genetics Unit (PNGU), Massachusetts General Hospital, Boston, MA, US
44, Research, 23andMe, Inc., Mountain View, CA, US
45, Neuroscience and Mental Health, Cardiff University, Cardiff, GB
46, Bioinformatics, University of British Columbia, Vancouver, BC, CA
47, Department of Epidemiology, Harvard T.H. Chan School of Public Health, Boston, MA, US
48, Department of Mathematics, Massachusetts Institute of Technology, Cambridge, MA, US
49, Department of Psychiatry (UPK), University of Basel, Basel, CH
50, Human Genomics Research Group, Department of Biomedicine, University of Basel, Basel, CH
51, Department of Genetic Epidemiology in Psychiatry, Central Institute of Mental Health, Medical Faculty Mannheim, Heidelberg University,

Mannheim, Baden-Württemberg, DE
52, Department of Psychiatry, Trinity College Dublin, Dublin, IE
53, Genetics, University of North Carolina at Chapel Hill, Chapel Hill, NC, US
54, Psychiatry & Behavioral Sciences, Johns Hopkins University, Baltimore, MD, US
55, Genetics and Computational Biology, QIMR Berghofer Medical Research Institute, Brisbane, QLD, AU
56, Bioinformatics Research Centre, Aarhus University, Aarhus, DK
57, Institute of Genetic Medicine, Newcastle University, Newcastle upon Tyne, GB
58, Danish Headache Centre, Department of Neurology, Rigshospitalet, Glostrup, DK
59, Institute of Biological Psychiatry, Mental Health Center Sct. Hans, Mental Health Services Capital Region of Denmark, Copenhagen, DK
60, iPSYCH, The Lundbeck Foundation Initiative for Psychiatric Research, Copenhagen, DK
61, Brain and Mind Centre, University of Sydney, Sydney, NSW, AU
62, Interfaculty Institute for Genetics and Functional Genomics, Department of Functional Genomics, University Medicine and Ernst Moritz
Arndt University Greifswald, Greifswald, Mecklenburg-Vorpommern, DE
63, Roche Pharmaceutical Research and Early Development, Pharmaceutical Sciences, Roche Innovation Center Basel, F. Hoffmann-La Roche
Ltd, Basel, CH
64, Quantitative Health Sciences, Cleveland Clinic, Cleveland, OH, US
65, Statistics, Pfizer Global Research and Development, Groton, CT, US
66, Max Planck Institute of Psychiatry, Munich, DE
67, Case Comprehensive Cancer Center, Case Western Reserve University, Cleveland, OH, US
68, Department of Genetics and Genome Sciences, Case Western Reserve University, Cleveland, OH, US
69, Division of Research, Kaiser Permanente Northern California, Oakland, CA, US
70, Psychiatry & The Behavioral Sciences, University of Southern California, Los Angeles, CA, US
71, Informatics Program, Boston Children's Hospital, Boston, MA, US
72, Department of Medicine, Brigham and Women's Hospital, Boston, MA, US
73, Department of Biomedical Informatics, Harvard Medical School, Boston, MA, US

74, Wellcome Trust Centre for Human Genetics, University of Oxford, Oxford, GB
75, Department of Endocrinology at Herlev University Hospital, University of Copenhagen, Copenhagen, DK
76, Swiss Institute of Bioinformatics, Lausanne, VD, CH
77, Institute of Social and Preventive Medicine (IUMSP), University Hospital of Lausanne, Lausanne, VD, CH
78, Dept of Anesthesia, Critical Care and Pain Medicine, Massachusetts General Hospital, Boston, MA, USA
79, Mental Health, NHS 24, Glasgow, GB
80, Division of Psychiatry, Centre for Clinical Brain Sciences, University of Edinburgh, Edinburgh, GB
81, Department of Psychiatry and Psychotherapy, University of Bonn, Bonn, DE
82, Statistics, University of Oxford, Oxford, GB
83, Psychiatry, Columbia University College of Physicians and Surgeons, New York, NY, US
84, School of Psychology and Counseling, Queensland University of Technology, Brisbane, QLD, AU
85, Child and Youth Mental Health Service, Children's Health Queensland Hospital and Health Service, South Brisbane, QLD, AU
86, Child Health Research Centre, University of Queensland, Brisbane, QLD, AU
87, Estonian Genome Center, University of Tartu, Tartu, EE
88, Medical Genetics, University of British Columbia, Vancouver, BC, CA
89, Statistics, University of British Columbia, Vancouver, BC, CA
90, DZHK (German Centre for Cardiovascular Research), Partner Site Greifswald, University Medicine, University Medicine Greifswald,
Greifswald, Mecklenburg-Vorpommern, DE
91, Institute of Clinical Chemistry and Laboratory Medicine, University Medicine Greifswald, Greifswald, Mecklenburg-Vorpommern, DE
92, Institute of Health and Biomedical Innovation, Queensland University of Technology, Brisbane, QLD, AU
93, Humus, Reykjavik, IS
94, MRC Centre for Neuropsychiatric Genetics and Genomics, Cardiff University, Cardiff, GB

95, Virginia Institute for Psychiatric & Behavioral Genetics, Virginia Commonwealth University, Richmond, VA, US
96, Complex Trait Genetics, Vrije Universiteit Amsterdam, Amsterdam, NL
97, Clinical Genetics, Vrije Universiteit Medical Center, Amsterdam, NL
98, Department of Psychiatry, Brigham and Women's Hospital, Boston, MA, US
99, Solid Biosciences, Boston, MA, US
100, Department of Psychiatry, Washington University in Saint Louis School of Medicine, Saint Louis, MO, US
101, Department of Biochemistry and Molecular Biology II, Institute of Neurosciences, Center for Biomedical Research, University of Granada,
Granada, ES
102, Department of Psychiatry, University of Groningen, University Medical Center Groningen, Groningen, NL
103, Department of Psychiatry and Psychotherapy, Medical Center of the University of Munich, Campus Innenstadt, Munich, DE
104, Institute of Psychiatric Phenomics and Genomics (IPPG), Medical Center of the University of Munich, Campus Innenstadt, Munich, DE
105, Division of Cancer Epidemiology and Genetics, National Cancer Institute, Bethesda, MD, US
106, Behavioral Health Services, Kaiser Permanente Washington, Seattle, WA, US
107, Faculty of Medicine, Department of Psychiatry, University of Iceland, Reykjavik, IS
108, School of Medicine and Dentistry, James Cook University, Townsville, QLD, AU
109, Institute of Health and Wellbeing, University of Glasgow, Glasgow, GB
110, deCODE Genetics / Amgen, Reykjavik, IS
111, Psychiatry & Human Behavior, University of Mississippi Medical Center, Jackson, MS, US
112, College of Biomedical and Life Sciences, Cardiff University, Cardiff, GB
113, Institute of Epidemiology and Social Medicine, University of Münster, Münster, Nordrhein-Westfalen, DE
114, Institute for Community Medicine, University Medicine Greifswald, Greifswald, Mecklenburg-Vorpommern, DE

115, KG Jebsen Centre for Psychosis Research, Norway Division of Mental Health and Addiction, Oslo University Hospital, Oslo, NO
116, Department of Psychiatry, University of California, San Diego, San Diego, CA, US
117, Medical Genetics Section, CGEM, IGMM, University of Edinburgh, Edinburgh, GB
118, Clinical Neurosciences, University of Cambridge, Cambridge, GB
119, Internal Medicine, Erasmus MC, Rotterdam, Zuid-Holland, NL
120, Roche Pharmaceutical Research and Early Development, Neuroscience, Ophthalmology and Rare Diseases Discovery & Translational
Medicine Area, Roche Innovation Center Basel, F. Hoffmann-La Roche Ltd, Basel, CH
121, Department of Psychiatry and Psychotherapy, University Medicine Greifswald, Greifswald, Mecklenburg-Vorpommern, DE
122, Department of Psychiatry, Leiden University Medical Center, Leiden, NL
123, Virginia Institute of Psychiatric & Behavioral Genetics, Virginia Commonwealth University, Richmond, VA, US
124, Computational Sciences Center of Emphasis, Pfizer Global Research and Development, Cambridge, MA, US
125, Institute for Molecular Bioscience; Queensland Brain Institute, The University of Queensland, Brisbane, QLD, AU
126, Department of Genetics, University of Groningen, University Medical Center Groningen, Groningen, NL
127, Department of Psychiatry, University of Münster, Münster, Nordrhein-Westfalen, DE
128, Institute of Neuroscience and Medicine (INM-1), Research Center Juelich, Juelich, DE
129, Institute of Medical Genetics and Pathology, University Hospital Basel, University of Basel, Basel, CH
130, Amsterdam Public Health Institute, Vrije Universiteit Medical Center, Amsterdam, NL
131, Centre for Integrative Biology, Università degli Studi di Trento, Trento, Trentino-Alto Adige, IT
132, Department of Psychiatry and Psychotherapy, Medical Center, Faculty of Medicine, University of Freiburg, Freiburg, Rheinland-Pfalz, DE
133, Psychiatry, Kaiser Permanente Northern California, San Francisco, CA, US

134, Medical Research Council Human Genetics Unit, Institute of Genetics and Molecular Medicine, University of Edinburgh, Edinburgh, GB
135, Centre for Addiction and Mental Health, Toronto, ON, CA
136, Department of Psychiatry, University of Toronto, Toronto, ON, CA
137, Division of Psychiatry, University College London, London, GB
138, Neuroscience Therapeutic Area, Janssen Research and Development, LLC, Titusville, NJ, US
139, Institute of Molecular and Cell Biology, University of Tartu, Tartu, EE
140, Psychosis Research Unit, Aarhus University Hospital, Risskov, Aarhus, DK
141, University of Liverpool, Liverpool, GB
142, Mental Health Center Copenhagen, Copenhagen Universtity Hospital, Copenhagen, DK
143, Human Genetics and Computational Biomedicine, Pfizer Global Research and Development, Groton, CT, US
144, Psychiatry, Harvard Medical School, Boston, MA, US
145, Psychiatry, University of Iowa, Iowa City, IA, US
146, Department of Psychiatry and Behavioral Sciences, Johns Hopkins University, Baltimore, MD, US
147, Human Genetics Branch, NIMH Division of Intramural Research Programs, Bethesda, MD, US
148, Department of Psychiatry and Psychotherapy, University Medical Center Göttingen, Goettingen, Niedersachsen, DE
149, Faculty of Medicine, University of Iceland, Reykjavik, IS
150, Child and Adolescent Psychiatry, Erasmus MC, Rotterdam, Zuid-Holland, NL
151, Psychiatry, Erasmus MC, Rotterdam, Zuid-Holland, NL
152, Psychiatry, Dalhousie University, Halifax, NS, CA
153, Division of Epidemiology, New York State Psychiatric Institute, New York, NY, US
154, Department of Clinical Medicine, University of Copenhagen, Copenhagen, DK
155, Human Genetics and Computational Biomedicine, Pfizer Global Research and Development, Cambridge, MA, US
156, Perelman School of Medicine, University of Pennsylvania, Philadelphia, PA, US
157, Department of Medical & Molecular Genetics, King's College London, London, GB

158, Psychiatry & Behavioral Sciences, Stanford University, Stanford, CA, US
159, NIHR BRC for Mental Health, King's College London, London, GB
160, Psychiatry, University of North Carolina at Chapel Hill, Chapel Hill, NC, US
