# Supplementary material for: Using genetic drug-target networks to develop new drug hypotheses for major depressive disorder
Source: Transl Psychiatry. 2019 Mar 15;9:117. doi: 10.1038/s41398-019-0451-4 (PMC6420656; doi:10.1038/s41398-019-0451-4)
Supplement: Supplementary file 3 — Supplementary Figures 1-15. [file 41398_2019_451_MOESM3_ESM.pdf]

Supplementary Figure 1: A03 – Drugs for functional gastrointestinal disorders

drug	$-\log_{10}(p)$
ALIZAPRIDE	3.48
OXYPHENCYCLIMINE	2.75
PROPANTHELINE	2.75
DIPHEMANIL	2.6
BROMOPRIDE	2.23
METHYLSCOPOLAMINE	2.22
GLYCOPYRRONIUM	2.15
HEXOCYCLIUM	2.12
MEPENZOLATE	2.12
DOMPERIDONE	2.01
DICYCLOVERINE	1.66
OTILONIUM_BROMIDE	1.63
MOXAVERINE	1.63
CLEBOPRIDE	1.47
OXYPHENONIUM	1.27
CISAPRIDE	1.25
ISOPROPAMIDE	1.21
HYOSCYAMINE	1.09
METHANTHELINE	0.93
METOCLOPRAMIDE	0.81
DROTAVERINE	0.74
CILANSETRON	0.73
TRIDIHEXETHYL	0.71
PAPAVERINE	0.68
TREPIBUTONE	0.61
ALVERINE	0.57
ALOSETRON	0.36

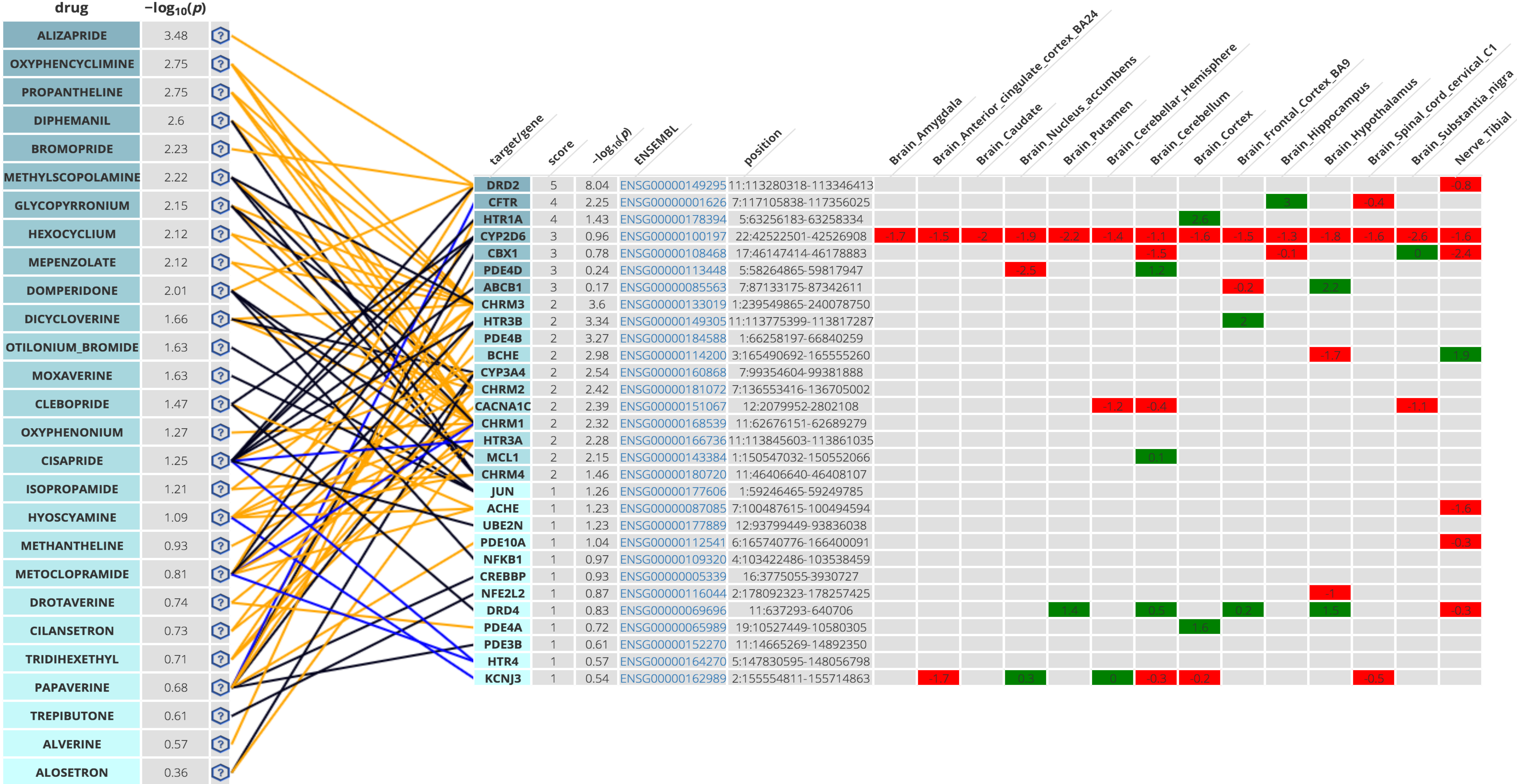

target/gene	score	$-\log_{10}(p)$	ENSEMBL	position	Brain_Amygdala	Brain_Anterior_cingulate_cortex_BA24	Brain_Caudate	Brain_Nucleus_accumbens	Brain_Putamen	Brain_Cerebellar_Hemisphere	Brain_Cerebellum	Brain_Cortex	Brain_Frontal_Cortex_BA9	Brain_Hippocampus	Brain_Hypothalamus	Brain_Spinal_cord_cervical_C1	Brain_Substantia_nigra	Nerve_Tibial
DRD2	5	8.04	ENSG00000149295	11:113280318-113346413														-0.8
CFTR	4	2.25	ENSG00000001626	7:117105838-117356025										3		-0.4		
HTR1A	4	1.43	ENSG00000178394	5:63256183-63258334								2.6						
CYP2D6	3	0.96	ENSG00000100197	22:42522501-42526908	-1.7	-1.5	-2	-1.9	-2.2	-1.4	-1.1	-1.6	-1.5	-1.3	-1.8	-1.6	-2.6	-1.6
CBX1	3	0.78	ENSG00000108468	17:46147414-46178883							-1.5			-0.1			0	-2.4
PDE4D	3	0.24	ENSG00000113448	5:58264865-59817947				-2.5			1.2							
ABCB1	3	0.17	ENSG00000085563	7:87133175-87342611									-0.2		2.2			
CHRM3	2	3.6	ENSG00000133019	1:239549865-240078750														
HTR3B	2	3.34	ENSG00000149305	11:113775399-113817287									2					
PDE4B	2	3.27	ENSG00000184588	1:66258197-66840259														
BCHE	2	2.98	ENSG00000114200	3:165490692-165555260											-1.7			1.9
CYP3A4	2	2.54	ENSG00000160868	7:99354604-99381888														
CHRM2	2	2.42	ENSG00000181072	7:136553416-136705002														
CACNA1C	2	2.39	ENSG00000151067	12:2079952-2802108						-1.2	-0.4						-1.1	
CHRM1	2	2.32	ENSG00000168539	11:62676151-62689279														
HTR3A	2	2.28	ENSG00000166736	11:113845603-113861035														
MCL1	2	2.15	ENSG00000143384	1:150547032-150552066							0.1							
CHRM4	2	1.46	ENSG00000180720	11:46406640-46408107														
JUN	1	1.26	ENSG00000177606	1:59246465-59249785														
ACHE	1	1.23	ENSG00000087085	7:100487615-100494594														-1.6
UBE2N	1	1.23	ENSG00000177889	12:93799449-93836038														
PDE10A	1	1.04	ENSG00000112541	6:165740776-166400091														-0.3
NFKB1	1	0.97	ENSG00000109320	4:103422486-103538459														
CREBBP	1	0.93	ENSG00000005339	16:3775055-3930727														
NFE2L2	1	0.87	ENSG00000116044	2:178092323-178257425											-1			
DRD4	1	0.83	ENSG00000069696	11:637293-640706					1.4		0.5		0.2		1.5			-0.3
PDE4A	1	0.72	ENSG00000065989	19:10527449-10580305								1.6						
PDE3B	1	0.61	ENSG00000152270	11:14665269-14892350														
HTR4	1	0.57	ENSG00000164270	5:147830595-148056798														
KCNJ3	1	0.54	ENSG00000162989	2:155554811-155714863		-1.7		0.3		0	-0.3	-0.2				-0.5		

— antagonist/inhibitor/negative modulator — partial agonist — agonist/activator/positive modulator — modulator — undefined bioactivity

Supplementary Figure 2: A03A – Drugs for functional gastrointestinal disorders

drug	$-\log_{10}(p)$
OXYPHENCYCLIMINE	2.75
PROPANTHELINE	2.75
DIPHEMANIL	2.6
GLYCOPYRRONIUM	2.15
HEXOCYCLIUM	2.12
MEPENZOLATE	2.12
PRIFINIUM_BROMIDE	2.09
DICYCLOVERINE	1.66
OTILONIUM_BROMIDE	1.63
MOXAVERINE	1.63
OXYPHENONIUM	1.27
ISOPROPAMIDE	1.21
METHANTHELINE	0.93
DROTAVERINE	0.74
CILANSETRON	0.73
TRIDIHEXETHYL	0.71
PAPAVERINE	0.68
TREPIBUTONE	0.61
ALVERINE	0.57
ALOSETRON	0.36
ISOMETHEPTENE	0.14

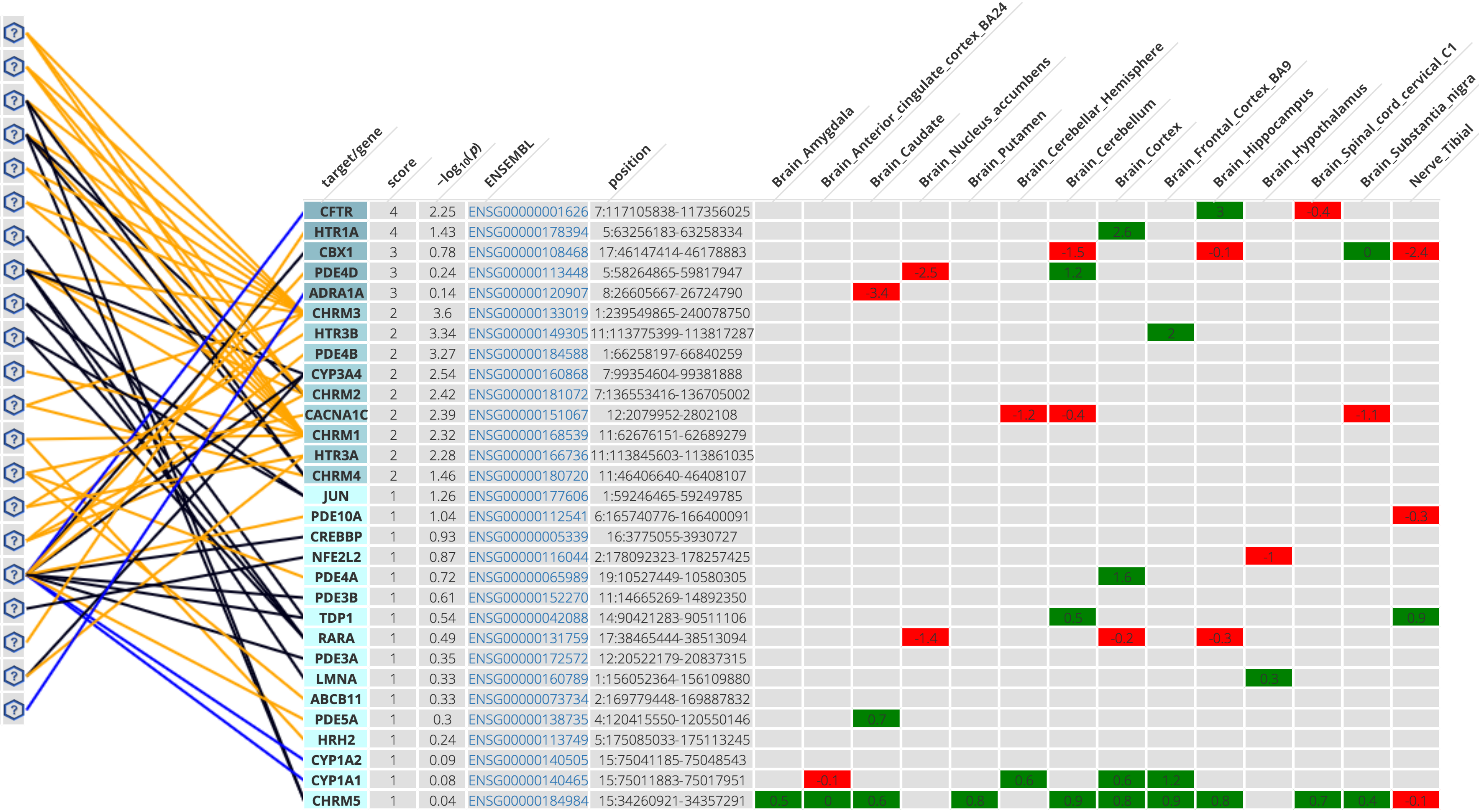

target/gene	score	$-\log_{10}(p)$	ENSEMBL	position
CFTR	4	2.25	ENSG00000001626	7:117105838-117356025
HTR1A	4	1.43	ENSG00000178394	5:63256183-63258334
CBX1	3	0.78	ENSG00000108468	17:46147414-46178883
PDE4D	3	0.24	ENSG00000113448	5:58264865-59817947
ADRA1A	3	0.14	ENSG00000120907	8:26605667-26724790
CHRM3	2	3.6	ENSG00000133019	1:239549865-240078750
HTR3B	2	3.34	ENSG00000149305	11:113775399-113817287
PDE4B	2	3.27	ENSG00000184588	1:66258197-66840259
CYP3A4	2	2.54	ENSG00000160868	7:99354604-99381888
CHRM2	2	2.42	ENSG00000181072	7:136553416-136705002
CACNA1C	2	2.39	ENSG00000151067	12:2079952-2802108
CHRM1	2	2.32	ENSG00000168539	11:62676151-62689279
HTR3A	2	2.28	ENSG00000166736	11:113845603-113861035
CHRM4	2	1.46	ENSG00000180720	11:46406640-46408107
JUN	1	1.26	ENSG00000177606	1:59246465-59249785
PDE10A	1	1.04	ENSG00000112541	6:165740776-166400091
CREBBP	1	0.93	ENSG00000005339	16:3775055-3930727
NFE2L2	1	0.87	ENSG00000116044	2:178092323-178257425
PDE4A	1	0.72	ENSG00000065989	19:10527449-10580305
PDE3B	1	0.61	ENSG00000152270	11:14665269-14892350
TDP1	1	0.54	ENSG00000042088	14:90421283-90511106
RARA	1	0.49	ENSG00000131759	17:38465444-38513094
PDE3A	1	0.35	ENSG00000172572	12:20522179-20837315
LMNA	1	0.33	ENSG00000160789	1:156052364-156109880
ABCB11	1	0.33	ENSG00000073734	2:169779448-169887832
PDE5A	1	0.3	ENSG00000138735	4:120415550-120550146
HRH2	1	0.24	ENSG00000113749	5:175085033-175113245
CYP1A2	1	0.09	ENSG00000140505	15:75041185-75048543
CYP1A1	1	0.08	ENSG00000140465	15:75011883-75017951
CHRM5	1	0.04	ENSG00000184984	15:34260921-34357291

target/gene	Brain_Amygdala	Brain_Anterior_cingulate_cortex_BA24	Brain_Caudate	Brain_Nucleus_accumbens	Brain_Putamen	Brain_Cerebellar_Hemisphere	Brain_Cerebellum	Brain_Cortex	Brain_Frontal_Cortex_BA9	Brain_Hippocampus	Brain_Hypothalamus	Brain_Spinal_cord_cervical_C1	Brain_Substantia_nigra	Nerve_Tibial
CFTR										3		-0.4		
HTR1A								2.6						
CBX1							-1.5			-0.1			0	-2.4
PDE4D				-2.5			1.2							
ADRA1A			-3.4											
CHRM3														
HTR3B									2					
PDE4B														
CYP3A4														
CHRM2														
CACNA1C						-1.2	-0.4						-1.1	
CHRM1														
HTR3A														
CHRM4														
JUN														
PDE10A														-0.3
CREBBP														
NFE2L2											-1			
PDE4A								1.6						
PDE3B														
TDP1							0.5							0.9
RARA				-1.4				-0.2		-0.3				
PDE3A														
LMNA											0.3			
ABCB11														
PDE5A			0.7											
HRH2														
CYP1A2														
CYP1A1		-0.1				0.6		0.6	1.2					
CHRM5	0.5	0	0.6		0.8		0.9	0.8	0.9	0.8		0.7	0.4	-0.1

— antagonist/inhibitor/negative modulator — partial agonist — agonist/activator/positive modulator — modulator — undefined bioactivity

Supplementary Figure 3: A03AB – Synthetic anticholinergics, quaternary ammonium compounds

drug	$-\log_{10}(p)$
PROPANTHELINE	2.75
DIPHEMANIL	2.6
GLYCOPYRRONIUM	2.15
HEXOCYCLIUM	2.12
MEPENZOLATE	2.12
PRIFINIUM_BROMIDE	2.09
OTILONIUM_BROMIDE	1.63
OXYPHENONIUM	1.27
ISOPROPAMIDE	1.21
METHANTHELINE	0.93
TRIDIHEXETHYL	0.71

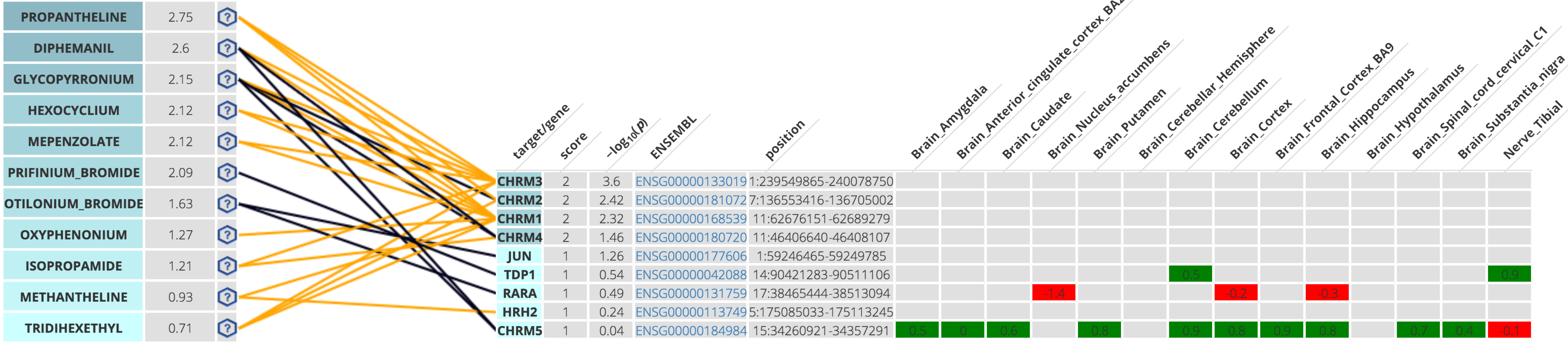

target/gene	score	$-\log_{10}(p)$	ENSEMBL	position	Brain_Amygdala	Brain_Anterior_cingulate_cortex_BA24	Brain_Caudate	Brain_Nucleus_accumbens	Brain_Putamen	Brain_Cerebellar_Hemisphere	Brain_Cerebellum	Brain_Cortex	Brain_Frontal_Cortex_BA9	Brain_Hippocampus	Brain_Hypothalamus	Brain_Spinal_cord_cervical_C1	Brain_Substantia_nigra	Nerve_Tibial
CHRM3	2	3.6	ENSG00000133019	1:239549865-240078750														
CHRM2	2	2.42	ENSG00000181072	7:136553416-136705002														
CHRM1	2	2.32	ENSG00000168539	11:62676151-62689279														
CHRM4	2	1.46	ENSG00000180720	11:46406640-46408107														
JUN	1	1.26	ENSG00000177606	1:59246465-59249785														
TDP1	1	0.54	ENSG00000042088	14:90421283-90511106							0.5							0.9
RARA	1	0.49	ENSG00000131759	17:38465444-38513094				-1.4				-0.2		-0.3				
HRH2	1	0.24	ENSG00000113749	5:175085033-175113245														
CHRM5	1	0.04	ENSG00000184984	15:34260921-34357291	0.5	0	0.6		0.8		0.9	0.8	0.9	0.8		0.7	0.4	-0.1

— antagonist/inhibitor/negative modulator — partial agonist — agonist/activator/positive modulator — modulator — undefined bioactivity

Supplementary Figure 4: C08 – Calcium channel blockers

drug	$-\log_{10}(p)$
ISRADIPINE	3.7
NITRENDIPINE	3.28
NICARDIPINE	2.73
LERCANIDIPINE	2.53
MIBEFRADIL	2.25
NIFEDIPINE	2.22
NILVADIPINE	2.17
NISOLDIPINE	2.16
FELODIPINE	2.1
LACIDIPINE	1.61
CILNIDIPINE	1.49
NIMODIPINE	1.47
BEPRIDIL	1.37
GALLOPAMIL	1.14
FENDILINE	0.82
CLEVIDIPINE	0.77
LIDOFLAZINE	0.75
VERAPAMIL	0.67
DILTIAZEM	0.58
MANIDIPINE	0.38
BENIDIPINE	0.38
AMLODIPINE	0.35

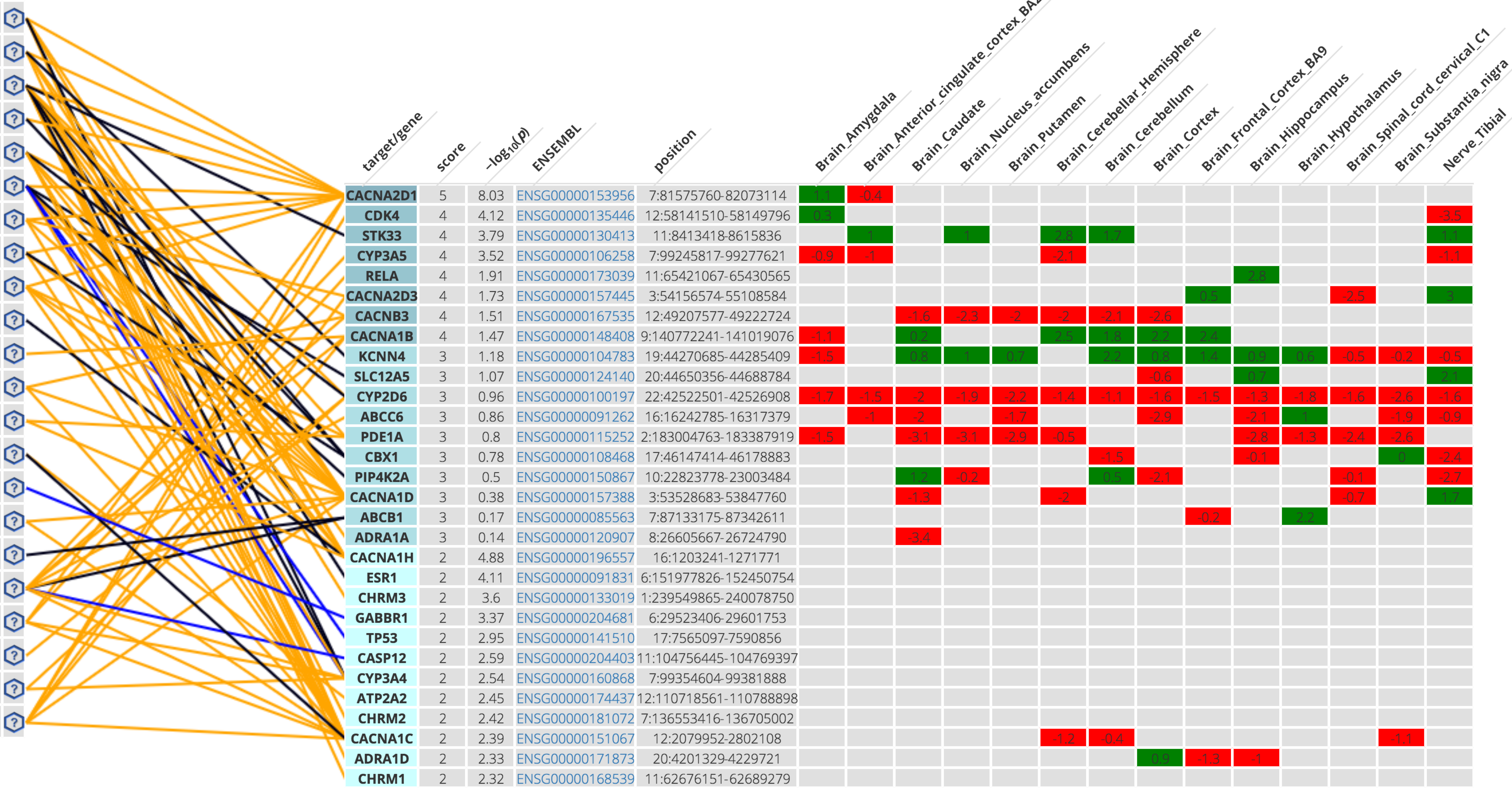

target/gene	score	$-\log_{10}(p)$	ENSEMBL	position	Brain_Amygdala	Brain_Anterior_cingulate_cortex_BA24	Brain_Caudate	Brain_Nucleus_accumbens	Brain_Putamen	Brain_Cerebellar_Hemisphere	Brain_Cerebellum	Brain_Cortex	Brain_Frontal_Cortex_BA9	Brain_Hippocampus	Brain_Hypothalamus	Brain_Spinal_cord_cervical_C1	Brain_Substantia_nigra	Nerve_Tibial
CACNA2D1	5	8.03	ENSG00000153956	7:81575760-82073114	1.1	-0.4												
CDK4	4	4.12	ENSG00000135446	12:58141510-58149796	0.3													-3.5
STK33	4	3.79	ENSG00000130413	11:8413418-8615836		1		1		2.8	1.7							1.1
CYP3A5	4	3.52	ENSG00000106258	7:99245817-99277621	-0.9	-1				-2.1								-1.1
RELA	4	1.91	ENSG00000173039	11:65421067-65430565										2.8				
CACNA2D3	4	1.73	ENSG00000157445	3:54156574-55108584									0.5			-2.5		3
CACNB3	4	1.51	ENSG00000167535	12:49207577-49222724			-1.6	-2.3	-2	-2	-2.1	-2.6						
CACNA1B	4	1.47	ENSG00000148408	9:140772241-141019076	-1.1		0.2			2.5	1.8	2.2	2.4					
KCNN4	3	1.18	ENSG00000104783	19:44270685-44285409	-1.5		0.8	1	0.7		2.2	0.8	1.4	0.9	0.6	-0.5	-0.2	-0.5
SLC12A5	3	1.07	ENSG00000124140	20:44650356-44688784								-0.6		0.7				2.1
CYP2D6	3	0.96	ENSG00000100197	22:42522501-42526908	-1.7	-1.5	-2	-1.9	-2.2	-1.4	-1.1	-1.6	-1.5	-1.3	-1.8	-1.6	-2.6	-1.6
ABCC6	3	0.86	ENSG00000091262	16:16242785-16317379		-1	-2		-1.7			-2.9		-2.1	1		-1.9	-0.9
PDE1A	3	0.8	ENSG00000115252	2:183004763-183387919	-1.5		-3.1	-3.1	-2.9	-0.5				-2.8	-1.3	-2.4	-2.6	
CBX1	3	0.78	ENSG00000108468	17:46147414-46178883							-1.5			-0.1			0	-2.4
PIP4K2A	3	0.5	ENSG00000150867	10:22823778-23003484			1.2	-0.2			0.5	-2.1				-0.1		-2.7
CACNA1D	3	0.38	ENSG00000157388	3:53528683-53847760			-1.3			-2						-0.7		1.7
ABCB1	3	0.17	ENSG00000085563	7:87133175-87342611									-0.2		2.2			
ADRA1A	3	0.14	ENSG00000120907	8:26605667-26724790			-3.4											
CACNA1H	2	4.88	ENSG00000196557	16:1203241-1271771														
ESR1	2	4.11	ENSG00000091831	6:151977826-152450754														
CHRM3	2	3.6	ENSG00000133019	1:239549865-240078750														
GABBR1	2	3.37	ENSG00000204681	6:29523406-29601753														
TP53	2	2.95	ENSG00000141510	17:7565097-7590856														
CASP12	2	2.59	ENSG00000204403	11:104756445-104769397														
CYP3A4	2	2.54	ENSG00000160868	7:99354604-99381888														
ATP2A2	2	2.45	ENSG00000174437	12:110718561-110788898														
CHRM2	2	2.42	ENSG00000181072	7:136553416-136705002														
CACNA1C	2	2.39	ENSG00000151067	12:2079952-2802108						-1.2	-0.4						-1.1	
ADRA1D	2	2.33	ENSG00000171873	20:4201329-4229721								0.9	-1.3	-1				
CHRM1	2	2.32	ENSG00000168539	11:62676151-62689279														

Supplementary Figure 5: C08C – Selective calcium channel blockers with mainly vascular effects

drug	$-\log_{10}(p)$
ISRADIPINE	3.7
NITRENDIPINE	3.28
NICARDIPINE	2.73
LERCANIDIPINE	2.53
MIBEFRADIL	2.25
NIFEDIPINE	2.22
NILVADIPINE	2.17
NISOLDIPINE	2.16
FELODIPINE	2.1
LACIDIPINE	1.61
CILNIDIPINE	1.49
NIMODIPINE	1.47
CLEVIDIPINE	0.77
MANIDIPINE	0.38
BENIDIPINE	0.38
AMLODIPINE	0.35

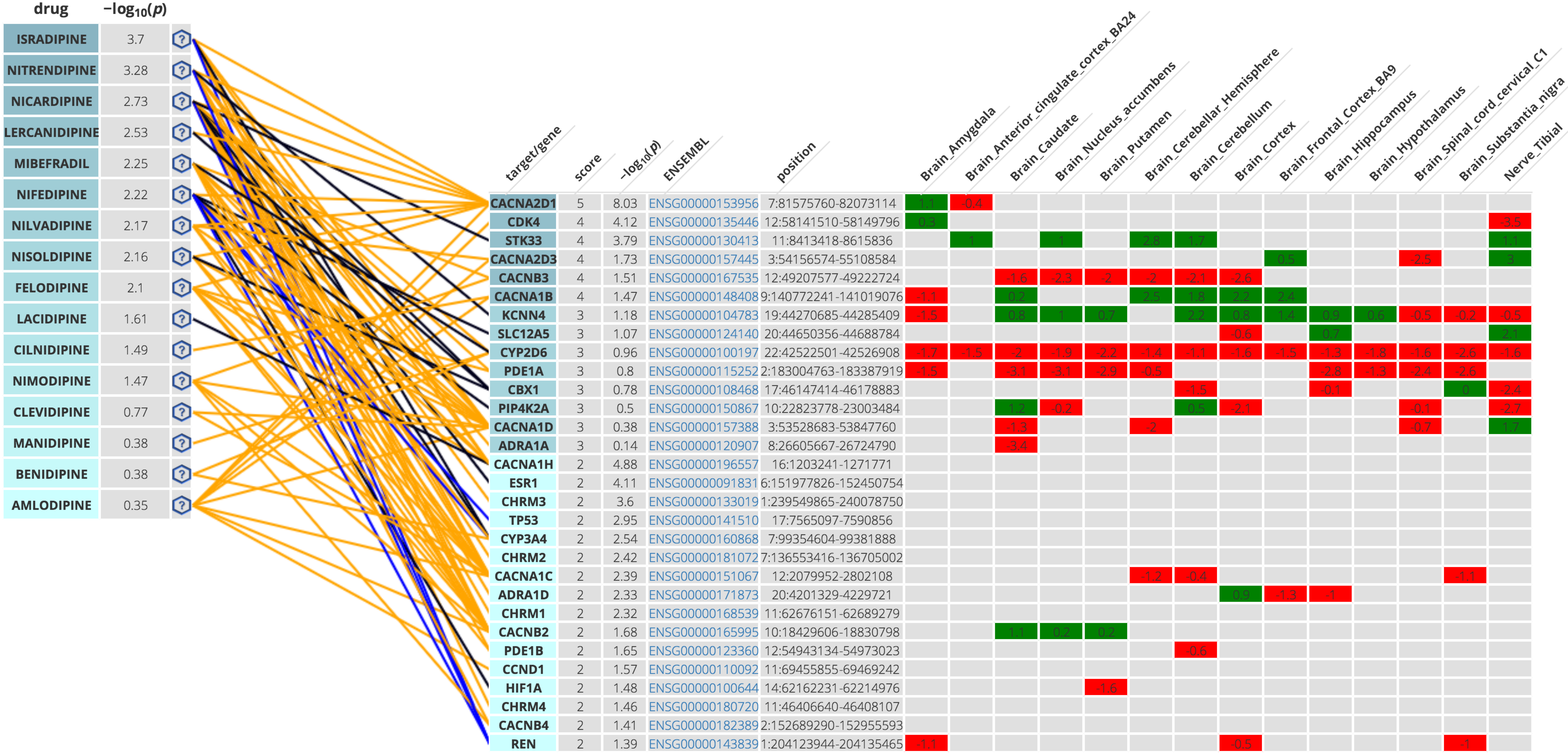

target/gene	score	$-\log_{10}(p)$	ENSEMBL	position	Brain_Amygdala	Brain_Anterior_cingulate_cortex_BA24	Brain_Caudate	Brain_Nucleus_accumbens	Brain_Putamen	Brain_Cerebellar_Hemisphere	Brain_Cerebellum	Brain_Cortex	Brain_Frontal_Cortex_BA9	Brain_Hippocampus	Brain_Hypothalamus	Brain_Spinal_cord_cervical_C1	Brain_Substantia_nigra	Nerve_Tibial
CACNA2D1	5	8.03	ENSG00000153956	7:81575760-82073114	1.1	-0.4												
CDK4	4	4.12	ENSG00000135446	12:58141510-58149796	0.3													-3.5
STK33	4	3.79	ENSG00000130413	11:8413418-8615836		1		1		2.8	1.7							1.1
CACNA2D3	4	1.73	ENSG00000157445	3:54156574-55108584									0.5			-2.5		3
CACNB3	4	1.51	ENSG00000167535	12:49207577-49222724			-1.6	-2.3	-2	-2	-2.1	-2.6						
CACNA1B	4	1.47	ENSG00000148408	9:140772241-141019076	-1.1		0.2			2.5	1.8	2.2	2.4					
KCNN4	3	1.18	ENSG00000104783	19:44270685-44285409	-1.5		0.8	1	0.7		2.2	0.8	1.4	0.9	0.6	-0.5	-0.2	-0.5
SLC12A5	3	1.07	ENSG00000124140	20:44650356-44688784								-0.6		0.7				2.1
CYP2D6	3	0.96	ENSG00000100197	22:42522501-42526908	-1.7	-1.5	-2	-1.9	-2.2	-1.4	-1.1	-1.6	-1.5	-1.3	-1.8	-1.6	-2.6	-1.6
PDE1A	3	0.8	ENSG00000115252	2:183004763-183387919	-1.5		-3.1	-3.1	-2.9	-0.5				-2.8	-1.3	-2.4	-2.6	
CBX1	3	0.78	ENSG00000108468	17:46147414-46178883							-1.5			-0.1			0	-2.4
PIP4K2A	3	0.5	ENSG00000150867	10:22823778-23003484			1.2	-0.2			0.5	-2.1				-0.1		-2.7
CACNA1D	3	0.38	ENSG00000157388	3:53528683-53847760			-1.3			-2						-0.7		1.7
ADRA1A	3	0.14	ENSG00000120907	8:26605667-26724790			-3.4											
CACNA1H	2	4.88	ENSG00000196557	16:1203241-1271771														
ESR1	2	4.11	ENSG00000091831	6:151977826-152450754														
CHRM3	2	3.6	ENSG00000133019	1:239549865-240078750														
TP53	2	2.95	ENSG00000141510	17:7565097-7590856														
CYP3A4	2	2.54	ENSG00000160868	7:99354604-99381888														
CHRM2	2	2.42	ENSG00000181072	7:136553416-136705002														
CACNA1C	2	2.39	ENSG00000151067	12:2079952-2802108						-1.2	-0.4						-1.1	
ADRA1D	2	2.33	ENSG00000171873	20:4201329-4229721								0.9	-1.3	-1				
CHRM1	2	2.32	ENSG00000168539	11:62676151-62689279														
CACNB2	2	1.68	ENSG00000165995	10:18429606-18830798			1.1	0.2	0.2									
PDE1B	2	1.65	ENSG00000123360	12:54943134-54973023							-0.6							
CCND1	2	1.57	ENSG00000110092	11:69455855-69469242														
HIF1A	2	1.48	ENSG00000100644	14:62162231-62214976					-1.6									
CHRM4	2	1.46	ENSG00000180720	11:46406640-46408107														
CACNB4	2	1.41	ENSG00000182389	2:152689290-152955593														
REN	2	1.39	ENSG00000143839	1:204123944-204135465	-1.1							-0.5					-1	

— antagonist/inhibitor/negative modulator — partial agonist — agonist/activator/positive modulator — modulator — undefined bioactivity

Supplementary Figure 6: C08CA – Dihydropyridine derivatives

drug	$-\log_{10}(p)$
ISRADIPINE	3.7
NITRENDIPINE	3.28
NICARDIPINE	2.73
LERCANIDIPINE	2.53
NIFEDIPINE	2.22
NILVADIPINE	2.17
NISOLDIPINE	2.16
FELODIPINE	2.1
LACIDIPINE	1.61
CILNIDIPINE	1.49
NIMODIPINE	1.47
CLEVIDIPINE	0.77
MANIDIPINE	0.38
BENIDIPINE	0.38
AMLODIPINE	0.35

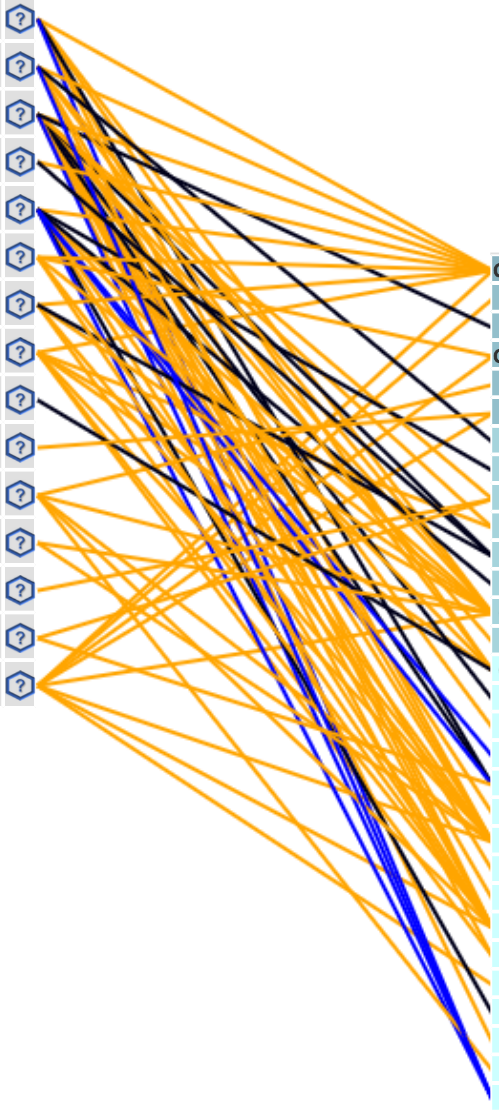

target/gene	score	$-\log_{10}(p)$	ENSEMBL	position	Brain_Amygdala	Brain_Anterior_cingulate_cortex_BA24	Brain_Caudate	Brain_Nucleus_accumbens	Brain_Putamen	Brain_Cerebellar_Hemisphere	Brain_Cerebellum	Brain_Cortex	Brain_Frontal_Cortex_BA9	Brain_Hippocampus	Brain_Hypothalamus	Brain_Spinal_cord_cervical_C1	Brain_Substantia_nigra	Nerve_Tibial
CACNA2D1	5	8.03	ENSG00000153956	7:81575760-82073114	1.1	-0.4												
CDK4	4	4.12	ENSG00000135446	12:58141510-58149796	0.3													-3.5
STK33	4	3.79	ENSG00000130413	11:8413418-8615836		1		1		2.8	1.7							1.1
CACNA2D3	4	1.73	ENSG00000157445	3:54156574-55108584									0.5			-2.5		3
CACNB3	4	1.51	ENSG00000167535	12:49207577-49222724			-1.6	-2.3	-2	-2	-2.1	-2.6						
CACNA1B	4	1.47	ENSG00000148408	9:140772241-141019076	-1.1		0.2			2.5	1.8	2.2	2.4					
KCNN4	3	1.18	ENSG00000104783	19:44270685-44285409	-1.5		0.8	1	0.7		2.2	0.8	1.4	0.9	0.6	-0.5	-0.2	-0.5
SLC12A5	3	1.07	ENSG00000124140	20:44650356-44688784								-0.6		0.7				2.1
CYP2D6	3	0.96	ENSG00000100197	22:42522501-42526908	-1.7	-1.5	-2	-1.9	-2.2	-1.4	-1.1	-1.6	-1.5	-1.3	-1.8	-1.6	-2.6	-1.6
PDE1A	3	0.8	ENSG00000115252	2:183004763-183387919	-1.5		-3.1	-3.1	-2.9	-0.5				-2.8	-1.3	-2.4	-2.6	
CBX1	3	0.78	ENSG00000108468	17:46147414-46178883							-1.5			-0.1			0	-2.4
PIP4K2A	3	0.5	ENSG00000150867	10:22823778-23003484			1.2	-0.2			0.5	-2.1				-0.1		-2.7
CACNA1D	3	0.38	ENSG00000157388	3:53528683-53847760			-1.3			-2						-0.7		1.7
ADRA1A	3	0.14	ENSG00000120907	8:26605667-26724790			-3.4											
CACNA1H	2	4.88	ENSG00000196557	16:1203241-1271771														
ESR1	2	4.11	ENSG00000091831	6:151977826-152450754														
CHRM3	2	3.6	ENSG00000133019	1:239549865-240078750														
TP53	2	2.95	ENSG00000141510	17:7565097-7590856														
CYP3A4	2	2.54	ENSG00000160868	7:99354604-99381888														
CHRM2	2	2.42	ENSG00000181072	7:136553416-136705002														
CACNA1C	2	2.39	ENSG00000151067	12:2079952-2802108						-1.2	-0.4						-1.1	
ADRA1D	2	2.33	ENSG00000171873	20:4201329-4229721								0.9	-1.3	-1				
CHRM1	2	2.32	ENSG00000168539	11:62676151-62689279														
CACNB2	2	1.68	ENSG00000165995	10:18429606-18830798			1.1	0.2	0.2									
PDE1B	2	1.65	ENSG00000123360	12:54943134-54973023							-0.6							
CCND1	2	1.57	ENSG00000110092	11:69455855-69469242														
HIF1A	2	1.48	ENSG00000100644	14:62162231-62214976					-1.6									
CHRM4	2	1.46	ENSG00000180720	11:46406640-46408107														
CACNB4	2	1.41	ENSG00000182389	2:152689290-152955593														
REN	2	1.39	ENSG00000143839	1:204123944-204135465	-1.1							-0.5					-1	

— antagonist/inhibitor/negative modulator — partial agonist — agonist/activator/positive modulator — modulator — undefined bioactivity

Supplementary Figure 7: G03 – Sex hormones and modulators of the genital system

drug	$-\log_{10}(p)$
LEVONORGESTREL	3.42
DIETHYLSTILBESTROL	3.22
METHYLTESTOSTERONE	3.13
GESTRINONE	3.13
DIENESTROL	2.2
NORETHISTERONE	2.17
LASOFOXIFENE	1.94
ETONOGESTREL	1.92
HYDROXYPROGESTERONE	1.92
CHLORMADINONE	1.61
MESTEROLONE	1.45
TIBOLONE	1.28
ESTRONE	1.27
ALLYLESTRENOL	1.2
FLUOXYMESTERONE	1.19
MOXESTROL	1.17
DROSPIRENONE	1.16
MEDROXYPROGESTERONE	1.15
ANDROSTANOLONE	1.04
DANAZOL	1.04
CONJUGATED_ESTROGENS	1.01
ETHINYLESTRADIOL	0.99
ESTRADIOL	0.96
LYNESTRENOL	0.74
CHLOROTRIANISENE	0.72
OSPEMIFENE	0.71
ETHISTERONE	0.65
TESTOSTERONE	0.59
MIFEPRISTONE	0.52

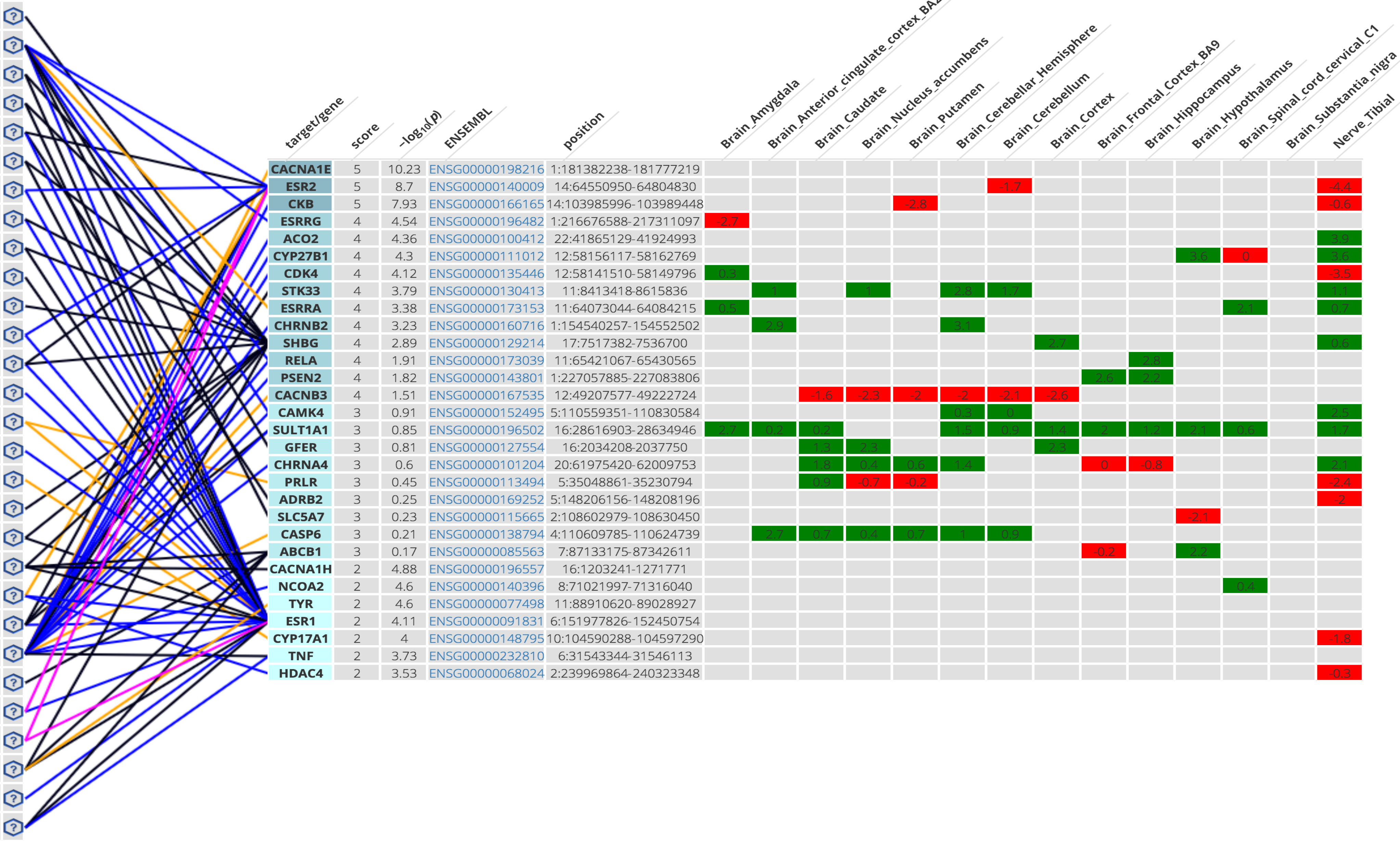

target/gene	score	$-\log_{10}(p)$	ENSEMBL	position	Brain_Amygdala	Brain_Anterior_cingulate_cortex_BA24	Brain_Caudate	Brain_Nucleus_accumbens	Brain_Putamen	Brain_Cerebellar_Hemisphere	Brain_Cerebellum	Brain_Cortex	Brain_Frontal_Cortex_BA9	Brain_Hippocampus	Brain_Hypothalamus	Brain_Spinal_cord_cervical_C1	Brain_Substantia_nigra	Nerve_Tibial
CACNA1E	5	10.23	ENSG00000198216	1:181382238-181777219														
ESR2	5	8.7	ENSG00000140009	14:64550950-64804830							-1.7							-4.4
CKB	5	7.93	ENSG00000166165	14:103985996-103989448					-2.8									-0.6
ESRRG	4	4.54	ENSG00000196482	1:216676588-217311097	-2.7													
ACO2	4	4.36	ENSG00000100412	22:41865129-41924993														3.9
CYP27B1	4	4.3	ENSG00000111012	12:58156117-58162769											3.6	0		3.6
CDK4	4	4.12	ENSG00000135446	12:58141510-58149796	0.3													-3.5
STK33	4	3.79	ENSG00000130413	11:8413418-8615836		1		1		2.8	1.7							1.1
ESRRA	4	3.38	ENSG00000173153	11:64073044-64084215	0.5											2.1		0.7
CHRNB2	4	3.23	ENSG00000160716	1:154540257-154552502		2.9				3.1								
SHBG	4	2.89	ENSG00000129214	17:7517382-7536700								2.7						0.6
RELA	4	1.91	ENSG00000173039	11:65421067-65430565										2.8				
PSEN2	4	1.82	ENSG00000143801	1:227057885-227083806									2.6	2.2				
CACNB3	4	1.51	ENSG00000167535	12:49207577-49222724			-1.6	-2.3	-2	-2	-2.1	-2.6						
CAMK4	3	0.91	ENSG00000152495	5:110559351-110830584						0.3	0							2.5
SULT1A1	3	0.85	ENSG00000196502	16:28616903-28634946	2.7	0.2	0.2			1.5	0.9	1.4	2	1.2	2.1	0.6		1.7
GFER	3	0.81	ENSG00000127554	16:2034208-2037750			1.3	2.3				2.3						
CHRNA4	3	0.6	ENSG00000101204	20:61975420-62009753			1.8	0.4	0.6	1.4			0	-0.8				2.1
PRLR	3	0.45	ENSG00000113494	5:35048861-35230794			0.9	-0.7	-0.2									-2.4
ADRB2	3	0.25	ENSG00000169252	5:148206156-148208196														-2
SLC5A7	3	0.23	ENSG00000115665	2:108602979-108630450											-2.1			
CASP6	3	0.21	ENSG00000138794	4:110609785-110624739		2.7	0.7	0.4	0.7	1	0.9							
ABCB1	3	0.17	ENSG00000085563	7:87133175-87342611									-0.2		2.2			
CACNA1H	2	4.88	ENSG00000196557	16:1203241-1271771														
NCOA2	2	4.6	ENSG00000140396	8:71021997-71316040												0.4		
TYR	2	4.6	ENSG00000077498	11:88910620-89028927														
ESR1	2	4.11	ENSG00000091831	6:151977826-152450754														
CYP17A1	2	4	ENSG00000148795	10:104590288-104597290														-1.8
TNF	2	3.73	ENSG00000232810	6:31543344-31546113														
HDAC4	2	3.53	ENSG00000068024	2:239969864-240323348														-0.3

— antagonist/inhibitor/negative modulator — partial agonist — agonist/activator/positive modulator — modulator — undefined bioactivity

Supplementary Figure 8: G03C – Estrogens

drug	$-\log_{10}(p)$
DIETHYLSTILBESTROL	3.22
DIENESTROL	2.2
TIBOLONE	1.28
ESTRONE	1.27
MOXESTROL	1.17
CONJUGATED_ESTROGENS	1.01
ETHINYLESTRADIOL	0.99
ESTRADIOL	0.96
CHLOROTRIANISENE	0.72
ESTRIOL	0.47

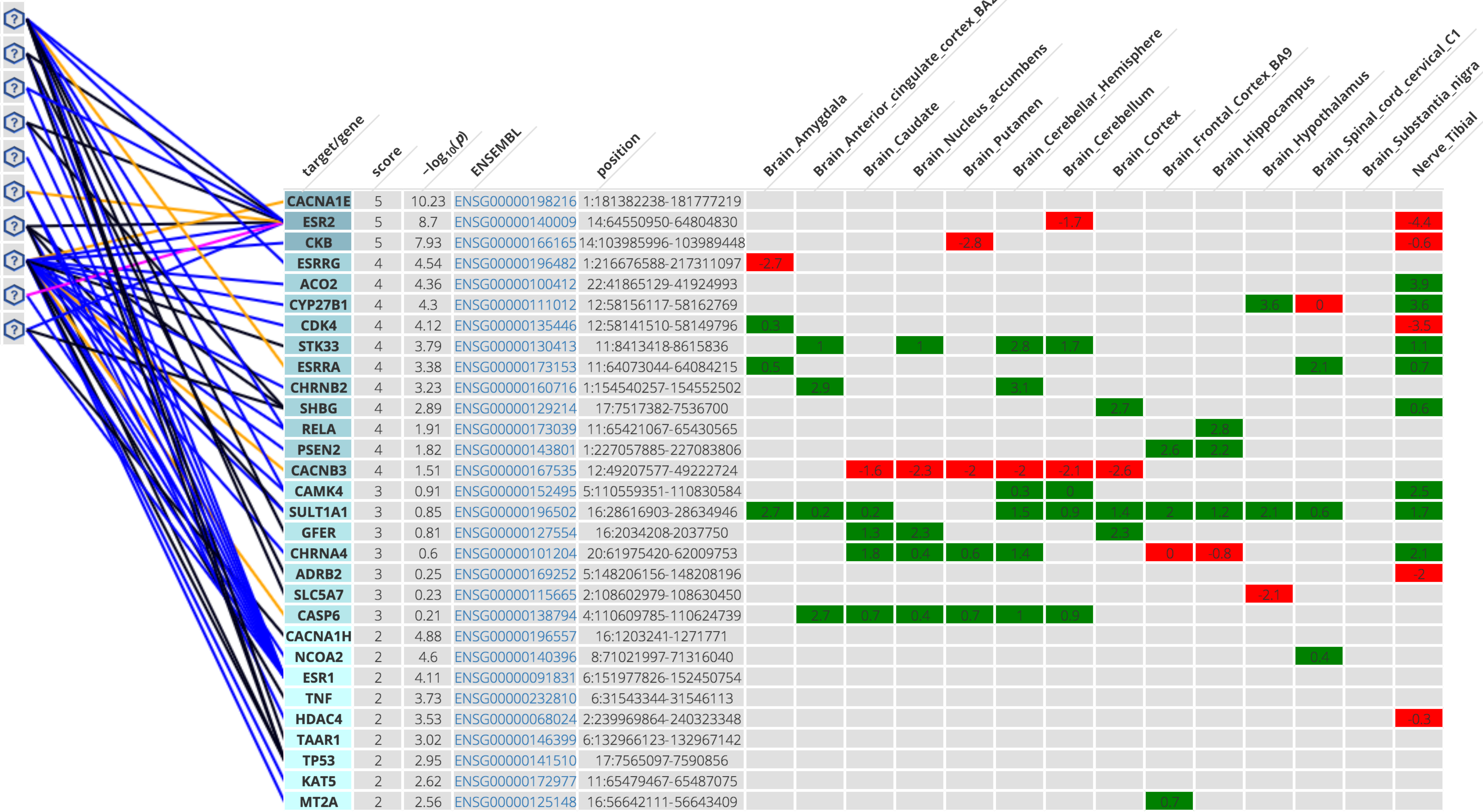

target/gene	score	$-\log_{10}(p)$	ENSEMBL	position	Brain_Amygdala	Brain_Anterior_cingulate_cortex_BA24	Brain_Caudate	Brain_Nucleus_accumbens	Brain_Putamen	Brain_Cerebellar_Hemisphere	Brain_Cerebellum	Brain_Cortex	Brain_Frontal_Cortex_BA9	Brain_Hippocampus	Brain_Hypothalamus	Brain_Spinal_cord_cervical_C1	Brain_Substantia_nigra	Nerve_Tibial
CACNA1E	5	10.23	ENSG00000198216	1:181382238-181777219														
ESR2	5	8.7	ENSG00000140009	14:64550950-64804830							-1.7							-4.4
CKB	5	7.93	ENSG00000166165	14:103985996-103989448					-2.8									-0.6
ESRRG	4	4.54	ENSG00000196482	1:216676588-217311097	-2.7													
ACO2	4	4.36	ENSG00000100412	22:41865129-41924993														3.9
CYP27B1	4	4.3	ENSG00000111012	12:58156117-58162769											3.6	0		3.6
CDK4	4	4.12	ENSG00000135446	12:58141510-58149796	0.3													-3.5
STK33	4	3.79	ENSG00000130413	11:8413418-8615836		1		1		2.8	1.7							1.1
ESRRA	4	3.38	ENSG00000173153	11:64073044-64084215	0.5											2.1		0.7
CHRNB2	4	3.23	ENSG00000160716	1:154540257-154552502		2.9				3.1								
SHBG	4	2.89	ENSG00000129214	17:7517382-7536700								2.7						0.6
RELA	4	1.91	ENSG00000173039	11:65421067-65430565										2.8				
PSEN2	4	1.82	ENSG00000143801	1:227057885-227083806									2.6	2.2				
CACNB3	4	1.51	ENSG00000167535	12:49207577-49222724			-1.6	-2.3	-2	-2	-2.1	-2.6						
CAMK4	3	0.91	ENSG00000152495	5:110559351-110830584						0.3	0							2.5
SULT1A1	3	0.85	ENSG00000196502	16:28616903-28634946	2.7	0.2	0.2			1.5	0.9	1.4	2	1.2	2.1	0.6		1.7
GFER	3	0.81	ENSG00000127554	16:2034208-2037750			1.3	2.3				2.3						
CHRNA4	3	0.6	ENSG00000101204	20:61975420-62009753			1.8	0.4	0.6	1.4			0	-0.8				2.1
ADRB2	3	0.25	ENSG00000169252	5:148206156-148208196														-2
SLC5A7	3	0.23	ENSG00000115665	2:108602979-108630450											-2.1			
CASP6	3	0.21	ENSG00000138794	4:110609785-110624739		2.7	0.7	0.4	0.7	1	0.9							
CACNA1H	2	4.88	ENSG00000196557	16:1203241-1271771														
NCOA2	2	4.6	ENSG00000140396	8:71021997-71316040												0.4		
ESR1	2	4.11	ENSG00000091831	6:151977826-152450754														
TNF	2	3.73	ENSG00000232810	6:31543344-31546113														
HDAC4	2	3.53	ENSG00000068024	2:239969864-240323348														-0.3
TAAR1	2	3.02	ENSG00000146399	6:132966123-132967142														
TP53	2	2.95	ENSG00000141510	17:7565097-7590856														
KAT5	2	2.62	ENSG00000172977	11:65479467-65487075														
MT2A	2	2.56	ENSG00000125148	16:56642111-56643409									0.7					

— antagonist/inhibitor/negative modulator — partial agonist — agonist/activator/positive modulator — modulator — undefined bioactivity

Supplementary Figure 9: N01A – Anesthetics, general

drug	$-\log_{10}(p)$
FENTANYL	2.42
ENFLURANE	1.9
SEVOFLURANE	1.89
HALOTHANE	1.87
ETOMIDATE	1.84
ISOFLURANE	1.56
PROPOFOL	1.37
DIETHYL_ETHER	1.27
REMIFENTANIL	1.15
DESFLURANE	1.07
KETAMINE	1
SUFENTANIL	0.87
ESKETAMINE	0.67
THIOPENTAL	0.63
TRICHLOROETHYLENE	0.47
HEXOBARBITAL	0.41
METHOHEXITAL	0.26
CHLOROFORM	0.13
NITROUS_OXIDE	0.12

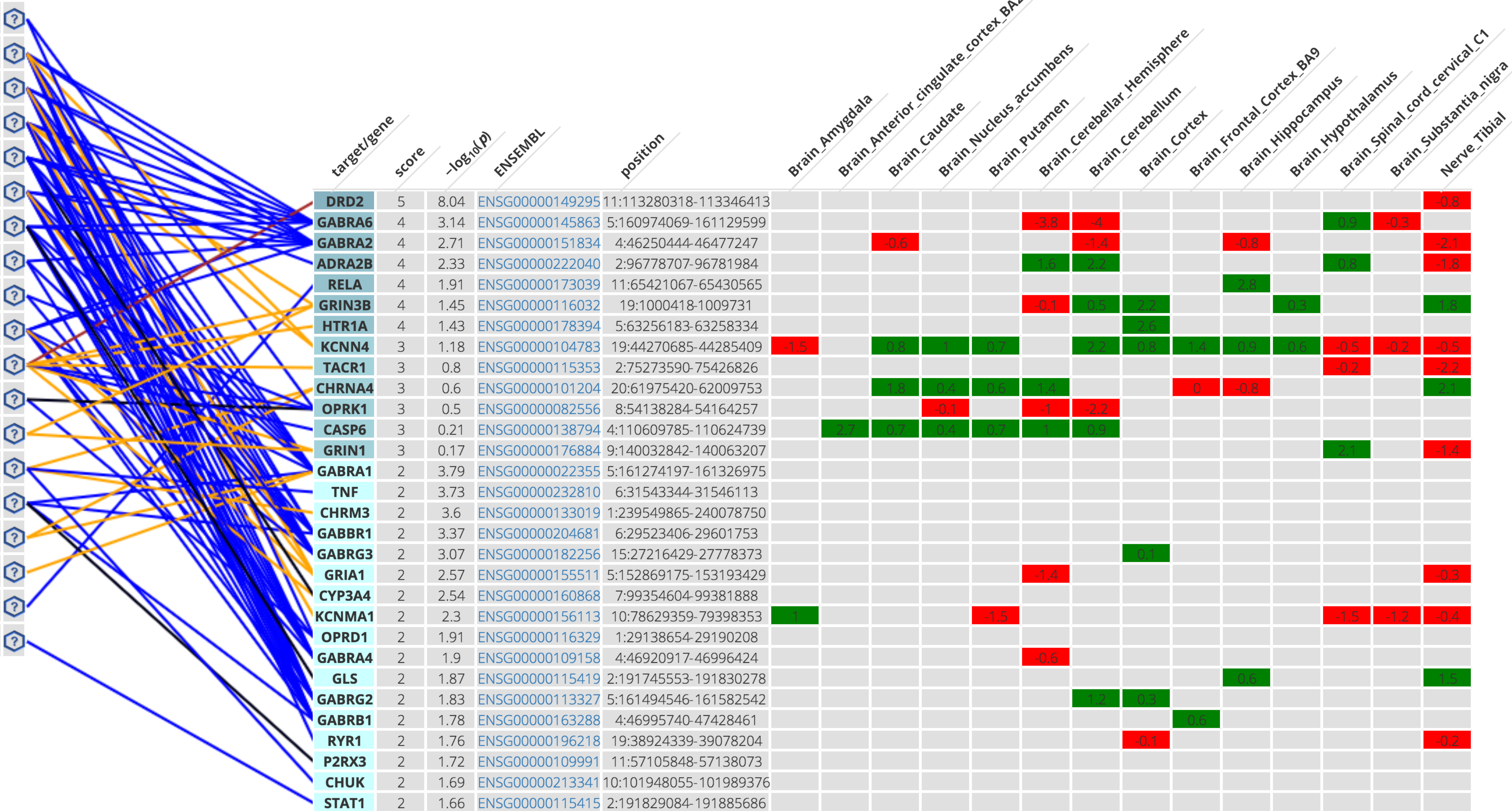

target/gene	score	$-\log_{10}(p)$	ENSEMBL	position	Brain_Amygdala	Brain_Anterior_cingulate_cortex_BA24	Brain_Caudate	Brain_Nucleus_accumbens	Brain_Putamen	Brain_Cerebellar_Hemisphere	Brain_Cerebellum	Brain_Cortex	Brain_Frontal_Cortex_BA9	Brain_Hippocampus	Brain_Hypothalamus	Brain_Spinal_cord_cervical_C1	Brain_Substantia_nigra	Nerve_Tibial
DRD2	5	8.04	ENSG00000149295	11:113280318-113346413														-0.8
GABRA6	4	3.14	ENSG00000145863	5:160974069-161129599						-3.8	-4					0.9	-0.3	
GABRA2	4	2.71	ENSG00000151834	4:46250444-46477247			-0.6				-1.4			-0.8				-2.1
ADRA2B	4	2.33	ENSG00000222040	2:96778707-96781984						1.6	2.2					0.8		-1.8
RELA	4	1.91	ENSG00000173039	11:65421067-65430565										2.8				
GRIN3B	4	1.45	ENSG00000116032	19:1000418-1009731						-0.1	0.5	2.2			0.3			1.8
HTR1A	4	1.43	ENSG00000178394	5:63256183-63258334								2.6						
KCNN4	3	1.18	ENSG00000104783	19:44270685-44285409	-1.5		0.8	1	0.7		2.2	0.8	1.4	0.9	0.6	-0.5	-0.2	-0.5
TACR1	3	0.8	ENSG00000115353	2:75273590-75426826												-0.2		-2.2
CHRNA4	3	0.6	ENSG00000101204	20:61975420-62009753			1.8	0.4	0.6	1.4			0	-0.8				2.1
OPRK1	3	0.5	ENSG00000082556	8:54138284-54164257				-0.1		-1	-2.2							
CASP6	3	0.21	ENSG00000138794	4:110609785-110624739		2.7	0.7	0.4	0.7	1	0.9							
GRIN1	3	0.17	ENSG00000176884	9:140032842-140063207												2.1		-1.4
GABRA1	2	3.79	ENSG00000022355	5:161274197-161326975														
TNF	2	3.73	ENSG00000232810	6:31543344-31546113														
CHRM3	2	3.6	ENSG00000133019	1:239549865-240078750														
GABBR1	2	3.37	ENSG00000204681	6:29523406-29601753														
GABRG3	2	3.07	ENSG00000182256	15:27216429-27778373								0.1						
GRIA1	2	2.57	ENSG00000155511	5:152869175-153193429						-1.4								-0.3
CYP3A4	2	2.54	ENSG00000160868	7:99354604-99381888														
KCNMA1	2	2.3	ENSG00000156113	10:78629359-79398353	1				-1.5							-1.5	-1.2	-0.4
OPRD1	2	1.91	ENSG00000116329	1:29138654-29190208														
GABRA4	2	1.9	ENSG00000109158	4:46920917-46996424						-0.6								
GLS	2	1.87	ENSG00000115419	2:191745553-191830278										0.6				1.5
GABRG2	2	1.83	ENSG00000113327	5:161494546-161582542							1.2	0.3						
GABRB1	2	1.78	ENSG00000163288	4:46995740-47428461									0.6					
RYR1	2	1.76	ENSG00000196218	19:38924339-39078204								-0.1						-0.2
P2RX3	2	1.72	ENSG00000109991	11:57105848-57138073														
CHUK	2	1.69	ENSG00000213341	10:101948055-101989376														
STAT1	2	1.66	ENSG00000115415	2:191829084-191885686														

Supplementary Figure 10: N04 – Anti-Parkinson drugs

drug	$-\log_{10}(p)$
PROCYCLIDINE	2.98
LEVODOPA	2.62
TRIHEXYPHENIDYL	2.4
BENZATROPINE	2.21
BIPERIDEN	2.14
ETANAUTINE	1.64
ORPHENADRINE_(CHLORIDE)	1.51
DEXETIMIDE	1.29
ROTIGOTINE	1.26
PIRIBEDIL	0.94
APOMORPHINE	0.91
PRAMIPEXOLE	0.77
ROPINIROLE	0.56
AMANTADINE	0.49
CABERGOLINE	0.47
PROFENAMINE	0.4
SELEGILINE	0.33
METIXENE	0.22
PERGOLIDE	0.21
BROMOCRIPTINE	0.17

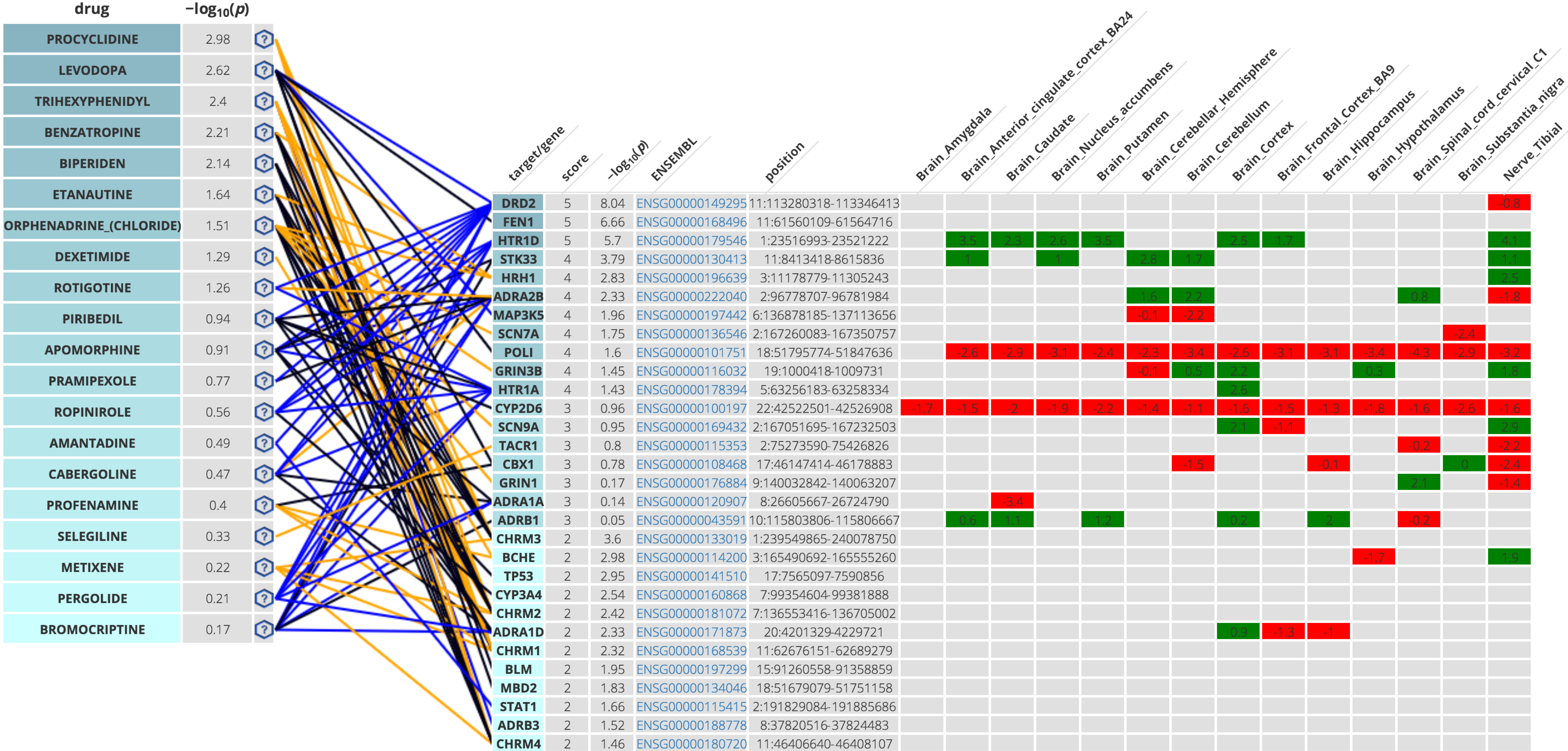

target/gene	score	$-\log_{10}(p)$	ENSEMBL	position	Brain_Amygdala	Brain_Anterior_cingulate_cortex_BA24	Brain_Caudate	Brain_Nucleus_accumbens	Brain_Putamen	Brain_Cerebellar_Hemisphere	Brain_Cerebellum	Brain_Cortex	Brain_Frontal_Cortex_BA9	Brain_Hippocampus	Brain_Hypothalamus	Brain_Spinal_cord_cervical_C1	Brain_Substantia_nigra	Nerve_Tibial
DRD2	5	8.04	ENSG00000149295	11:113280318-113346413														-0.8
FEN1	5	6.66	ENSG00000168496	11:61560109-61564716														
HTR1D	5	5.7	ENSG00000179546	1:23516993-23521222		3.5	2.3	2.6	3.5			2.5	1.7					4.1
STK33	4	3.79	ENSG00000130413	11:8413418-8615836		1		1		2.8	1.7							1.1
HRH1	4	2.83	ENSG00000196639	3:11178779-11305243														2.5
ADRA2B	4	2.33	ENSG00000222040	2:96778707-96781984						1.6	2.2					0.8		-1.8
MAP3K5	4	1.96	ENSG00000197442	6:136878185-137113656						-0.1	-2.2							
SCN7A	4	1.75	ENSG00000136546	2:167260083-167350757													-2.4	
POLI	4	1.6	ENSG00000101751	18:51795774-51847636		-2.6	-2.9	-3.1	-2.4	-2.3	-3.4	-2.6	-3.1	-3.1	-3.4	-4.3	-2.9	-3.2
GRIN3B	4	1.45	ENSG00000116032	19:1000418-1009731						-0.1	0.5	2.2			0.3			1.8
HTR1A	4	1.43	ENSG00000178394	5:63256183-63258334								2.6						
CYP2D6	3	0.96	ENSG00000100197	22:42522501-42526908	-1.7	-1.5	-2	-1.9	-2.2	-1.4	-1.1	-1.6	-1.5	-1.3	-1.8	-1.6	-2.6	-1.6
SCN9A	3	0.95	ENSG00000169432	2:167051695-167232503								2.1	-1.1					2.9
TACR1	3	0.8	ENSG00000115353	2:75273590-75426826												-0.2		-2.2
CBX1	3	0.78	ENSG00000108468	17:46147414-46178883							-1.5			-0.1			0	-2.4
GRIN1	3	0.17	ENSG00000176884	9:140032842-140063207												2.1		-1.4
ADRA1A	3	0.14	ENSG00000120907	8:26605667-26724790			-3.4											
ADRB1	3	0.05	ENSG00000043591	10:115803806-115806667		0.6	1.1		1.2			0.2		2		-0.2		
CHRM3	2	3.6	ENSG00000133019	1:239549865-240078750														
BCHE	2	2.98	ENSG00000114200	3:165490692-165555260											-1.7			1.9
TP53	2	2.95	ENSG00000141510	17:7565097-7590856														
CYP3A4	2	2.54	ENSG00000160868	7:99354604-99381888														
CHRM2	2	2.42	ENSG00000181072	7:136553416-136705002														
ADRA1D	2	2.33	ENSG00000171873	20:4201329-4229721								0.9	-1.3	-1				
CHRM1	2	2.32	ENSG00000168539	11:62676151-62689279														
BLM	2	1.95	ENSG00000197299	15:91260558-91358859														
MBD2	2	1.83	ENSG00000134046	18:51679079-51751158														
STAT1	2	1.66	ENSG00000115415	2:191829084-191885686														
ADRB3	2	1.52	ENSG00000188778	8:37820516-37824483														
CHRM4	2	1.46	ENSG00000180720	11:46406640-46408107														

— antagonist/inhibitor/negative modulator — partial agonist — agonist/activator/positive modulator — modulator — undefined bioactivity

Supplementary Figure 11: N05 – Psycholeptics

drug	$-\log_{10}(p)$
MESORIDAZINE	4.39
BROTIZOLAM	2.91
PROPIOMAZINE	2.67
THIORIDAZINE	2.57
CLOTIAPINE	2.4
SCOPOLAMINE	2.31
ZUCLOPENTHIXOL	2.14
OXAZEPAM	2.03
FLUSPIRILENE	1.92
CHLORPROETHAZINE	1.9
MOPERONE	1.9
PIMOZIDE	1.88
CYAMEMAZINE	1.87
LEVOSULPIRIDE	1.81
ZIPRASIDONE	1.79
FLUPHENAZINE	1.76
ACETOPHENAZINE	1.76
PROCHLORPERAZINE	1.71
ALPRAZOLAM	1.71
ZOTEPINE	1.64
MEPROBAMATE	1.51
TRIAZOLAM	1.47
QUAZEPAM	1.47
GLUTETHIMIDE	1.47
METHYPRYLON	1.47
ETHCHLORVYNOL	1.47
LORAZEPAM	1.47
PRAZEPAM	1.47

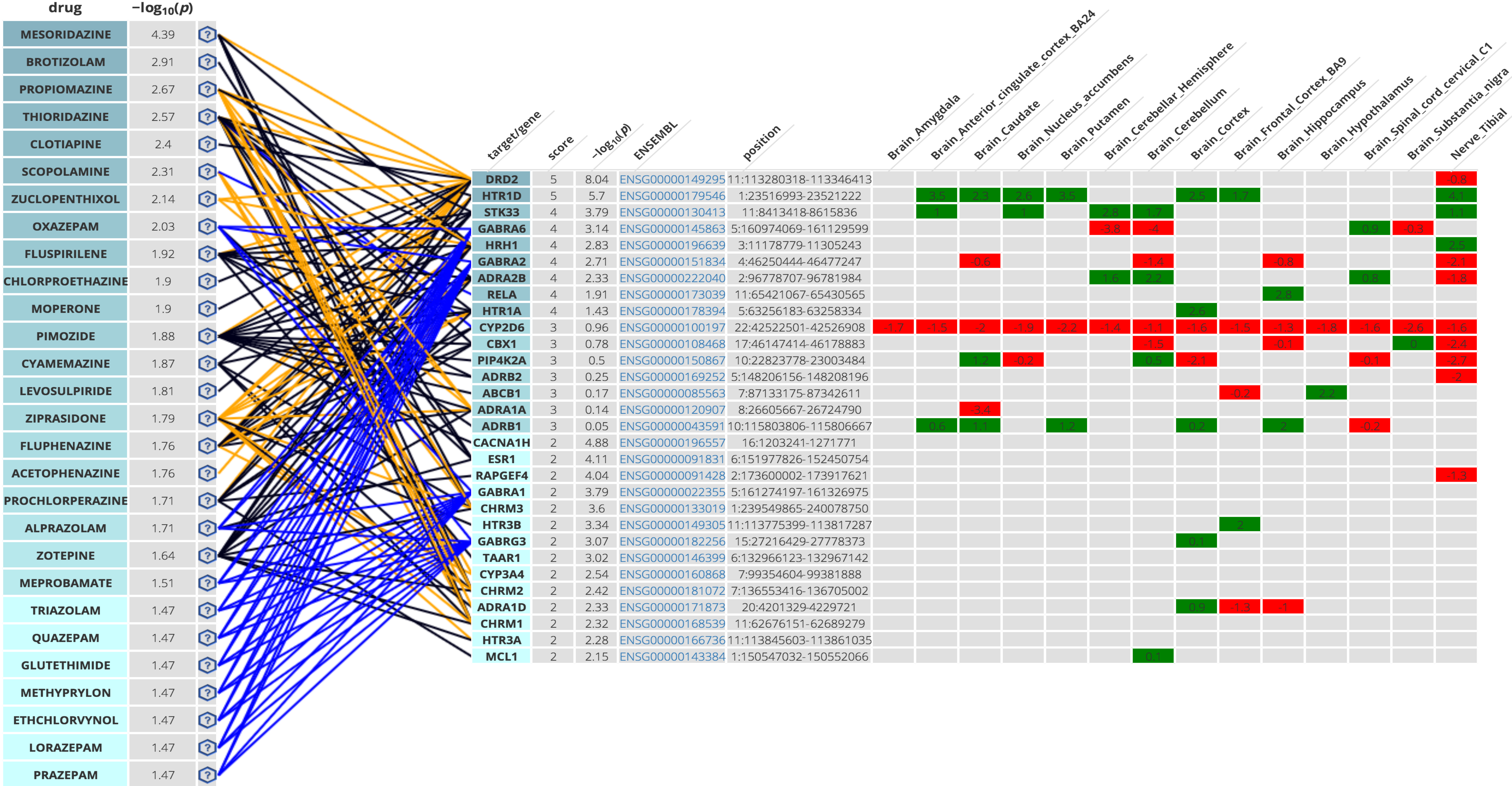

target/gene	score	$-\log_{10}(p)$	ENSEMBL	position	Brain_Amygdala	Brain_Anterior_cingulate_cortex_BA24	Brain_Caudate	Brain_Nucleus_accumbens	Brain_Putamen	Brain_Cerebellar_Hemisphere	Brain_Cerebellum	Brain_Cortex	Brain_Frontal_Cortex_BA9	Brain_Hippocampus	Brain_Hypothalamus	Brain_Spinal_cord_cervical_C1	Brain_Substantia_nigra	Nerve_Tibial
DRD2	5	8.04	ENSG00000149295	11:113280318-113346413														-0.8
HTR1D	5	5.7	ENSG00000179546	1:23516993-23521222		3.5	2.3	2.6	3.5			2.5	1.7					4.1
STK33	4	3.79	ENSG00000130413	11:8413418-8615836		1		1		2.8	1.7							1.1
GABRA6	4	3.14	ENSG00000145863	5:160974069-161129599						-3.8	-4					0.9	-0.3	
HRH1	4	2.83	ENSG00000196639	3:11178779-11305243														2.5
GABRA2	4	2.71	ENSG00000151834	4:46250444-46477247			-0.6				-1.4			-0.8				-2.1
ADRA2B	4	2.33	ENSG00000222040	2:96778707-96781984						1.6	2.2					0.8		-1.8
RELA	4	1.91	ENSG00000173039	11:65421067-65430565										2.8				
HTR1A	4	1.43	ENSG00000178394	5:63256183-63258334								2.6						
CYP2D6	3	0.96	ENSG00000100197	22:42522501-42526908	-1.7	-1.5	-2	-1.9	-2.2	-1.4	-1.1	-1.6	-1.5	-1.3	-1.8	-1.6	-2.6	-1.6
CBX1	3	0.78	ENSG00000108468	17:46147414-46178883							-1.5			-0.1			0	-2.4
PIP4K2A	3	0.5	ENSG00000150867	10:22823778-23003484			1.2	-0.2			0.5	-2.1				-0.1		-2.7
ADRB2	3	0.25	ENSG00000169252	5:148206156-148208196														-2
ABCB1	3	0.17	ENSG00000085563	7:87133175-87342611									-0.2		2.2			
ADRA1A	3	0.14	ENSG00000120907	8:26605667-26724790			3.4											
ADRB1	3	0.05	ENSG00000043591	10:115803806-115806667		0.6	1.1		1.2			0.2		2		-0.2		
CACNA1H	2	4.88	ENSG00000196557	16:1203241-1271771														
ESR1	2	4.11	ENSG00000091831	6:151977826-152450754														
RAPGEF4	2	4.04	ENSG00000091428	2:173600002-173917621														-1.3
GABRA1	2	3.79	ENSG00000022355	5:161274197-161326975														
CHRM3	2	3.6	ENSG00000133019	1:239549865-240078750														
HTR3B	2	3.34	ENSG00000149305	11:113775399-113817287									2					
GABRG3	2	3.07	ENSG00000182256	15:27216429-27778373								0.1						
TAAR1	2	3.02	ENSG00000146399	6:132966123-132967142														
CYP3A4	2	2.54	ENSG00000160868	7:99354604-99381888														
CHRM2	2	2.42	ENSG00000181072	7:136553416-136705002														
ADRA1D	2	2.33	ENSG00000171873	20:4201329-4229721								0.9	-1.3	-1				
CHRM1	2	2.32	ENSG00000168539	11:62676151-62689279														
HTR3A	2	2.28	ENSG00000166736	11:113845603-113861035														
MCL1	2	2.15	ENSG00000143384	1:150547032-150552066							0.1							

— antagonist/inhibitor/negative modulator — partial agonist — agonist/activator/positive modulator — modulator — undefined bioactivity

Supplementary Figure 12: N05A – Antipsychotics

drug	$-\log_{10}(p)$
MESORIDAZINE	4.39
THIORIDAZINE	2.57
CLOTIAPINE	2.4
ZUCLOPENTHIXOL	2.14
FLUSPIRILENE	1.92
CHLORPROETHAZINE	1.9
MOPERONE	1.9
PIMOZIDE	1.88
CYAMEMAZINE	1.87
LEVOSULPIRIDE	1.81
ZIPRASIDONE	1.79
FLUPHENAZINE	1.76
ACETOPHENAZINE	1.76
PROCHLORPERAZINE	1.71
ZOTEPINE	1.64
FLUPENTIXOL	1.41
OLANZAPINE	1.35
SERTINDOLE	1.35
CHLORPROTHIXENE	1.34
BREXPIPRAZOLE	1.28
ARIPIPRAZOLE	1.27
SULTOPRIDE	1.24
QUETIAPINE	1.23
PROMAZINE	1.22
LEVOMEPROMAZINE	1.13
CARIPRAZINE	1.05
SULPIRIDE	0.97
CHLORPROMAZINE	0.96

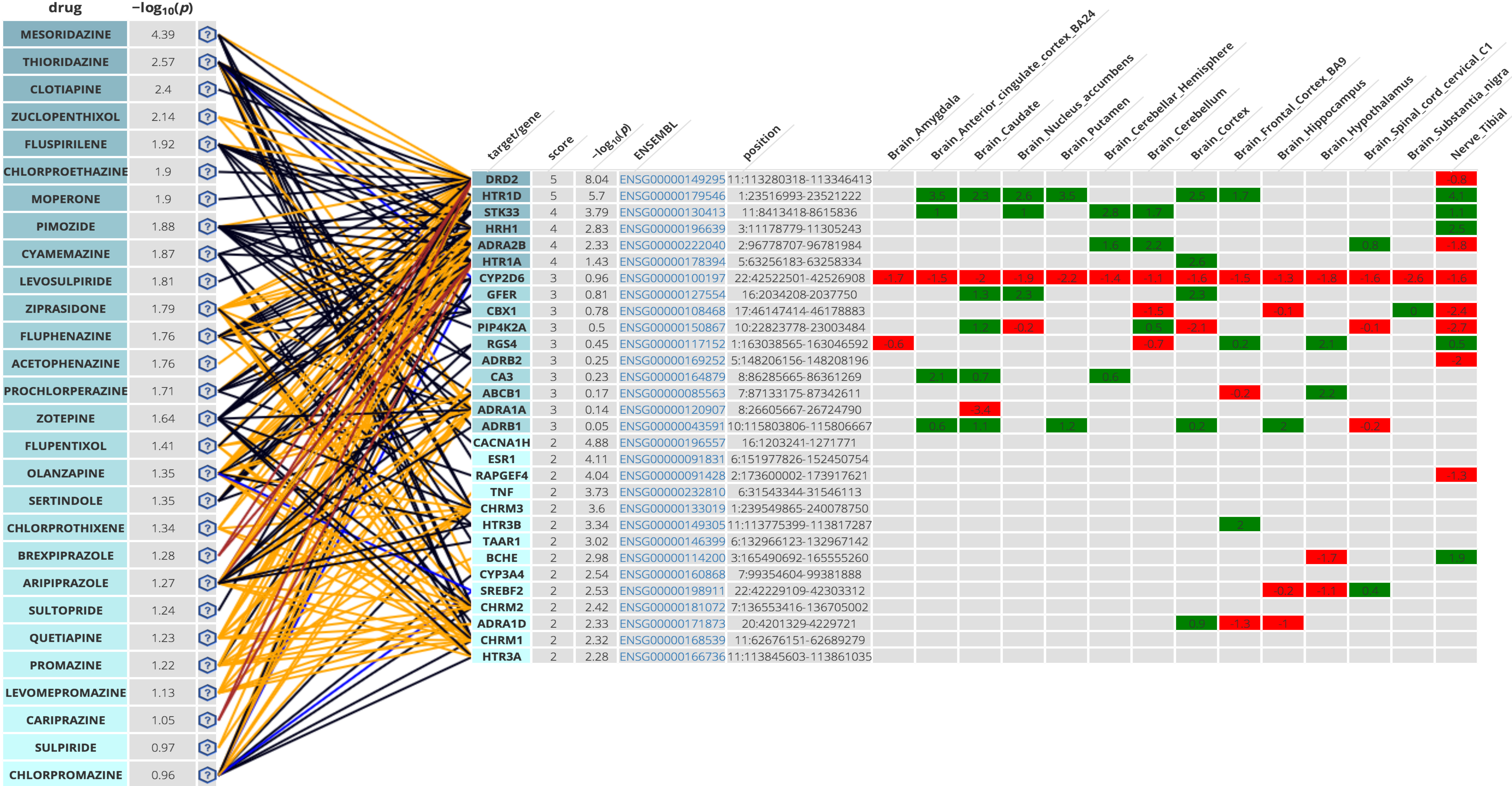

target/gene	score	$-\log_{10}(p)$	ENSEMBL	position	Brain_Amygdala	Brain_Anterior_cingulate_cortex_BA24	Brain_Caudate	Brain_Nucleus_accumbens	Brain_Putamen	Brain_Cerebellar_Hemisphere	Brain_Cerebellum	Brain_Cortex	Brain_Frontal_Cortex_BA9	Brain_Hippocampus	Brain_Hypothalamus	Brain_Spinal_cord_cervical_C1	Brain_Substantia_nigra	Nerve_Tibial
DRD2	5	8.04	ENSG00000149295	11:113280318-113346413														-0.8
HTR1D	5	5.7	ENSG00000179546	1:23516993-23521222		3.5	2.3	2.6	3.5			2.5	1.7					4.1
STK33	4	3.79	ENSG00000130413	11:8413418-8615836		1		1		2.8	1.7							1.1
HRH1	4	2.83	ENSG00000196639	3:11178779-11305243														2.5
ADRA2B	4	2.33	ENSG00000222040	2:96778707-96781984						1.6	2.2					0.8		-1.8
HTR1A	4	1.43	ENSG00000178394	5:63256183-63258334								2.6						
CYP2D6	3	0.96	ENSG00000100197	22:42522501-42526908	-1.7	-1.5	-2	-1.9	-2.2	-1.4	-1.1	-1.6	-1.5	-1.3	-1.8	-1.6	-2.6	-1.6
GFER	3	0.81	ENSG00000127554	16:2034208-2037750			1.3	2.3				2.3						
CBX1	3	0.78	ENSG00000108468	17:46147414-46178883							-1.5			-0.1			0	-2.4
PIP4K2A	3	0.5	ENSG00000150867	10:22823778-23003484			1.2	-0.2			0.5	-2.1				-0.1		-2.7
RGS4	3	0.45	ENSG00000117152	1:163038565-163046592	-0.6						-0.7		0.2		2.1			0.5
ADRB2	3	0.25	ENSG00000169252	5:148206156-148208196														-2
CA3	3	0.23	ENSG00000164879	8:86285665-86361269		2.1	0.7			0.6								
ABCB1	3	0.17	ENSG00000085563	7:87133175-87342611									-0.2		2.2			
ADRA1A	3	0.14	ENSG00000120907	8:26605667-26724790			-3.4											
ADRB1	3	0.05	ENSG00000043591	10:115803806-115806667		0.6	1.1		1.2			0.2		2		-0.2		
CACNA1H	2	4.88	ENSG00000196557	16:1203241-1271771														
ESR1	2	4.11	ENSG00000091831	6:151977826-152450754														
RAPGEF4	2	4.04	ENSG00000091428	2:173600002-173917621														-1.3
TNF	2	3.73	ENSG00000232810	6:31543344-31546113														
CHRM3	2	3.6	ENSG00000133019	1:239549865-240078750														
HTR3B	2	3.34	ENSG00000149305	11:113775399-113817287									2					
TAAR1	2	3.02	ENSG00000146399	6:132966123-132967142														
BCHE	2	2.98	ENSG00000114200	3:165490692-165555260											-1.7			1.9
CYP3A4	2	2.54	ENSG00000160868	7:99354604-99381888														
SREBF2	2	2.53	ENSG00000198911	22:42229109-42303312										-0.2	-1.1	0.4		
CHRM2	2	2.42	ENSG00000181072	7:136553416-136705002														
ADRA1D	2	2.33	ENSG00000171873	20:4201329-4229721								0.9	-1.3	-1				
CHRM1	2	2.32	ENSG00000168539	11:62676151-62689279														
HTR3A	2	2.28	ENSG00000166736	11:113845603-113861035														

— antagonist/inhibitor/negative modulator — partial agonist — agonist/activator/positive modulator — modulator — undefined bioactivity

Supplementary Figure 13: N06AA – Non-selective monoamine reuptake inhibitors

drug	$-\log_{10}(p)$
DOSULEPIN	2.11
TRIMIPRAMINE	1.9
DESIPRAMINE	1.81
LOFEPRAMINE	1.75
AMOXAPINE	0.99
AMITRIPTYLINE	0.98
NORTRIPTYLINE	0.94
DOXEPIN	0.91
IPRINDOLE	0.75
AMINEPTINE	0.73
CLOMIPRAMINE	0.67
IMIPRAMINE	0.64
BUTRIPTYLINE	0.61
DIMETACRINE	0.58
PROTRIPTYLINE	0.55
MAPROTILINE	0.52

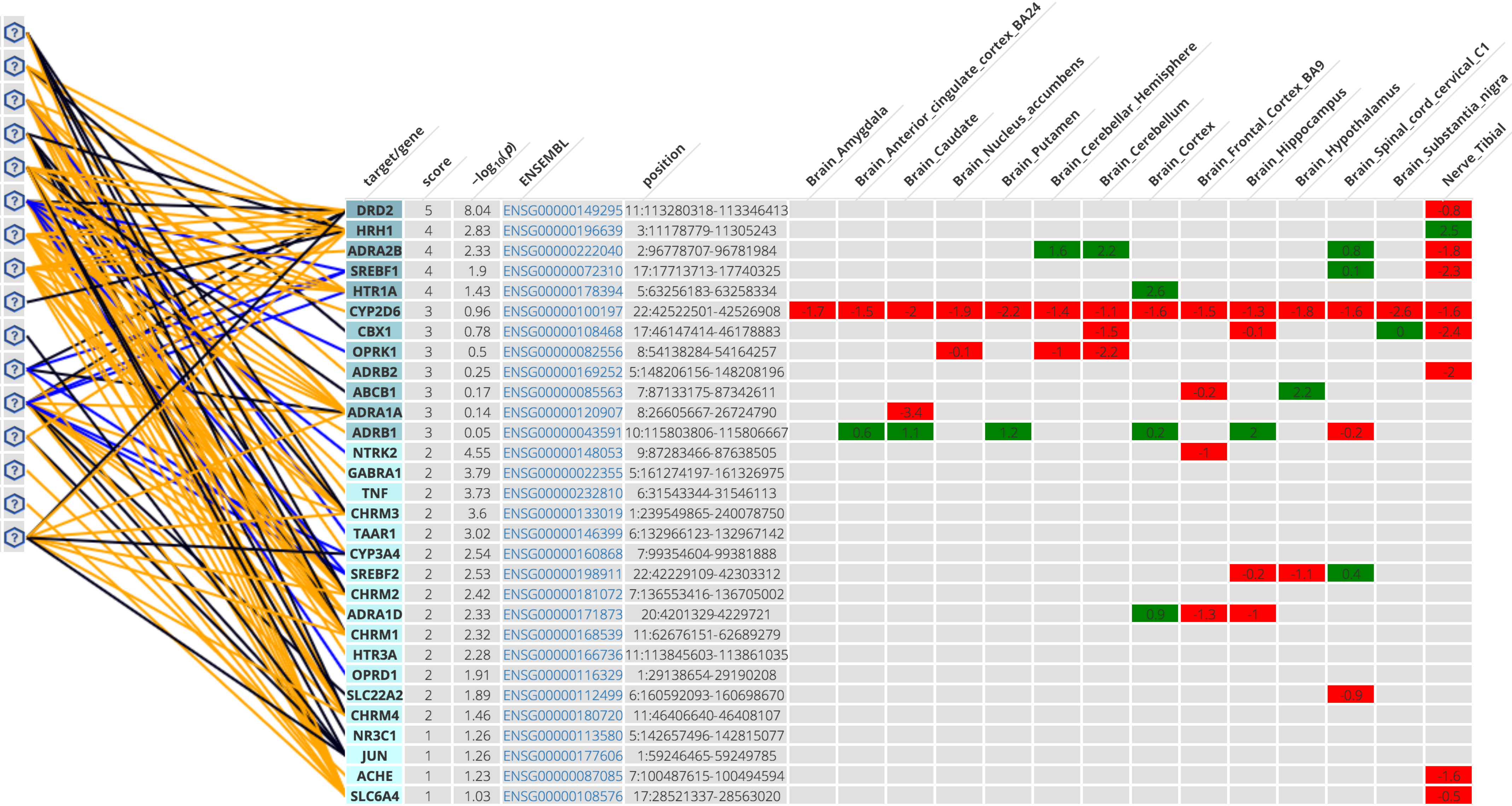

target/gene	score	$-\log_{10}(p)$	ENSEMBL	position	Brain_Amygdala	Brain_Anterior_cingulate_cortex_BA24	Brain_Caudate	Brain_Nucleus_accumbens	Brain_Putamen	Brain_Cerebellar_Hemisphere	Brain_Cerebellum	Brain_Cortex	Brain_Frontal_Cortex_BA9	Brain_Hippocampus	Brain_Hypothalamus	Brain_Spinal_cord_cervical_C1	Brain_Substantia_nigra	Nerve_Tibial
DRD2	5	8.04	ENSG00000149295	11:113280318-113346413														-0.8
HRH1	4	2.83	ENSG00000196639	3:11178779-11305243														2.5
ADRA2B	4	2.33	ENSG00000222040	2:96778707-96781984						1.6	2.2					0.8		-1.8
SREBF1	4	1.9	ENSG00000072310	17:17713713-17740325												0.1		-2.3
HTR1A	4	1.43	ENSG00000178394	5:63256183-63258334								2.6						
CYP2D6	3	0.96	ENSG00000100197	22:42522501-42526908	-1.7	-1.5	-2	-1.9	-2.2	-1.4	-1.1	-1.6	-1.5	-1.3	-1.8	-1.6	-2.6	-1.6
CBX1	3	0.78	ENSG00000108468	17:46147414-46178883							-1.5			-0.1			0	2.4
OPRK1	3	0.5	ENSG00000082556	8:54138284-54164257				-0.1		-1	-2.2							
ADRB2	3	0.25	ENSG00000169252	5:148206156-148208196														-2
ABCB1	3	0.17	ENSG00000085563	7:87133175-87342611									-0.2		2.2			
ADRA1A	3	0.14	ENSG00000120907	8:26605667-26724790			-3.4											
ADRB1	3	0.05	ENSG00000043591	10:115803806-115806667		0.6	1.1		1.2			0.2		2		-0.2		
NTRK2	2	4.55	ENSG00000148053	9:87283466-87638505									-1					
GABRA1	2	3.79	ENSG00000022355	5:161274197-161326975														
TNF	2	3.73	ENSG00000232810	6:31543344-31546113														
CHRM3	2	3.6	ENSG00000133019	1:239549865-240078750														
TAAR1	2	3.02	ENSG00000146399	6:132966123-132967142														
CYP3A4	2	2.54	ENSG00000160868	7:99354604-99381888														
SREBF2	2	2.53	ENSG00000198911	22:42229109-42303312										-0.2	-1.1	0.4		
CHRM2	2	2.42	ENSG00000181072	7:136553416-136705002														
ADRA1D	2	2.33	ENSG00000171873	20:4201329-4229721								0.9	-1.3	-1				
CHRM1	2	2.32	ENSG00000168539	11:62676151-62689279														
HTR3A	2	2.28	ENSG00000166736	11:113845603-113861035														
OPRD1	2	1.91	ENSG00000116329	1:29138654-29190208														
SLC22A2	2	1.89	ENSG00000112499	6:160592093-160698670												-0.9		
CHRM4	2	1.46	ENSG00000180720	11:46406640-46408107														
NR3C1	1	1.26	ENSG00000113580	5:142657496-142815077														
JUN	1	1.26	ENSG00000177606	1:59246465-59249785														
ACHE	1	1.23	ENSG00000087085	7:100487615-100494594														-1.6
SLC6A4	1	1.03	ENSG00000108576	17:28521337-28563020														-0.5

— antagonist/inhibitor/negative modulator — partial agonist — agonist/activator/positive modulator — modulator — undefined bioactivity

Supplementary Figure 14: R06A – Antihistamines for systemic use

drug	$-\log_{10}(p)$
BROMPHENIRAMINE	3.48
CHLORPHENAMINE	3.11
PROMETHAZINE	2.2
CARBINOXAMINE	2
MEQUITAZINE	1.96
TRIPELENNAMINE	1.87
ACRIVASTINE	1.82
OXATOMIDE	1.79
BROMAZINE	1.73
AZATADINE	1.6
TERFENADINE	1.55
BUCLIZINE	1.49
DIMETINDENE	1.43
ASTEMIZOLE	1.41
DIPHENYLPYRALINE	1.41
RUPATADINE	1.38
EBASTINE	1.21
CYPROHEPTADINE	1.19
LORATADINE	1.05
CETIRIZINE	1.03
MIZOLASTINE	1
DESLORATADINE	0.98
MEPYRAMINE	0.95
ANTAZOLINE	0.9
FEXOFENADINE	0.87
THIETHYLPERAZINE	0.87
ALIMEMAZINE	0.81

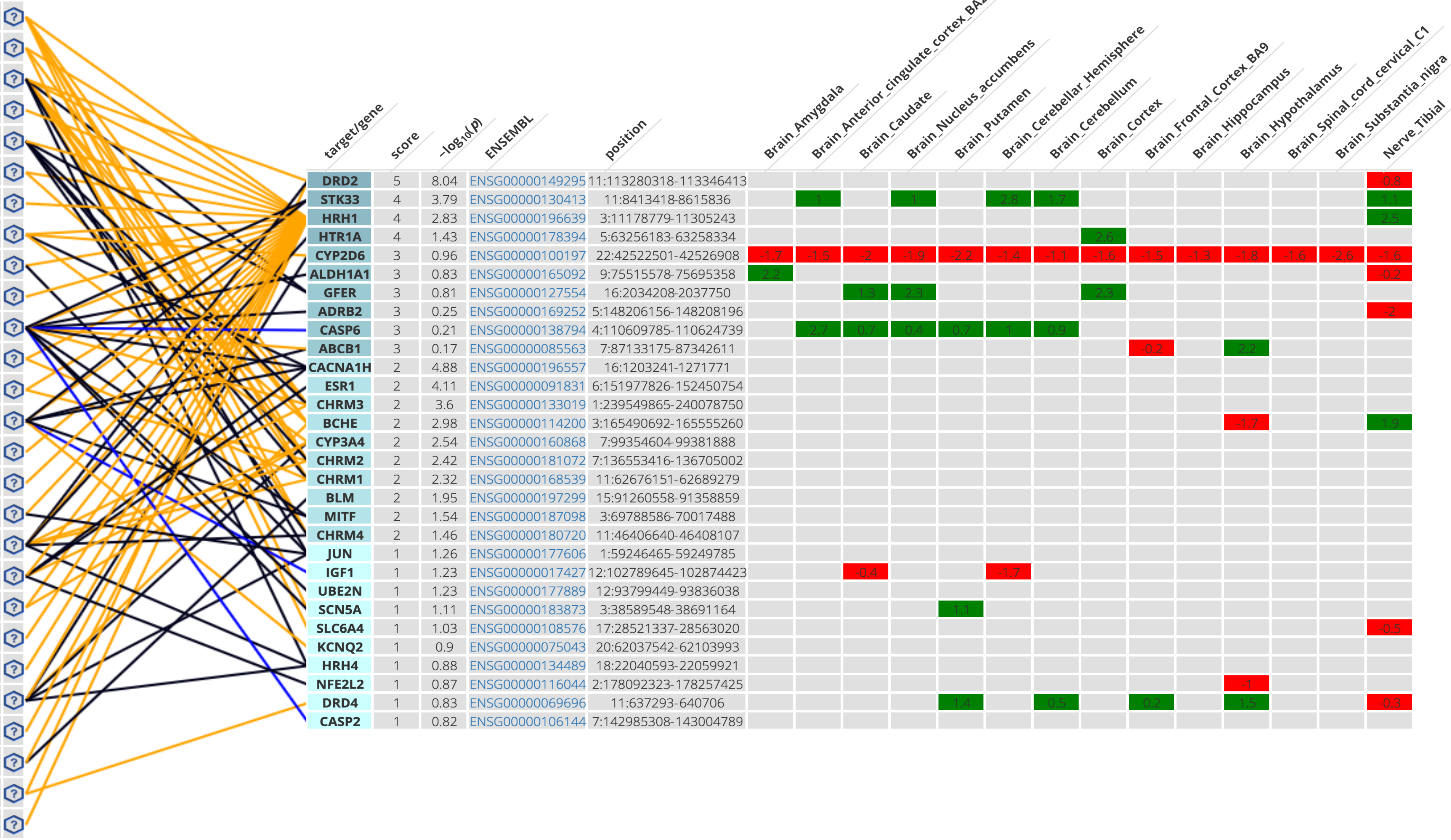

target/gene	score	$-\log_{10}(p)$	ENSEMBL	position
DRD2	5	8.04	ENSG00000149295	11:113280318-113346413
STK33	4	3.79	ENSG00000130413	11:8413418-8615836
HRH1	4	2.83	ENSG00000196639	3:11178779-11305243
HTR1A	4	1.43	ENSG00000178394	5:63256183-63258334
CYP2D6	3	0.96	ENSG00000100197	22:42522501-42526908
ALDH1A1	3	0.83	ENSG00000165092	9:75515578-75695358
GFER	3	0.81	ENSG00000127554	16:2034208-2037750
ADRB2	3	0.25	ENSG00000169252	5:148206156-148208196
CASP6	3	0.21	ENSG00000138794	4:110609785-110624739
ABCB1	3	0.17	ENSG00000085563	7:87133175-87342611
CACNA1H	2	4.88	ENSG00000196557	16:1203241-1271771
ESR1	2	4.11	ENSG00000091831	6:151977826-152450754
CHRM3	2	3.6	ENSG00000133019	1:239549865-240078750
BCHE	2	2.98	ENSG00000114200	3:165490692-165555260
CYP3A4	2	2.54	ENSG00000160868	7:99354604-99381888
CHRM2	2	2.42	ENSG00000181072	7:136553416-136705002
CHRM1	2	2.32	ENSG00000168539	11:62676151-62689279
BLM	2	1.95	ENSG00000197299	15:91260558-91358859
MITF	2	1.54	ENSG00000187098	3:69788586-70017488
CHRM4	2	1.46	ENSG00000180720	11:46406640-46408107
JUN	1	1.26	ENSG00000177606	1:59246465-59249785
IGF1	1	1.23	ENSG00000017427	12:102789645-102874423
UBE2N	1	1.23	ENSG00000177889	12:93799449-93836038
SCN5A	1	1.11	ENSG00000183873	3:38589548-38691164
SLC6A4	1	1.03	ENSG00000108576	17:28521337-28563020
KCNQ2	1	0.9	ENSG00000075043	20:62037542-62103993
HRH4	1	0.88	ENSG00000134489	18:22040593-22059921
NFE2L2	1	0.87	ENSG00000116044	2:178092323-178257425
DRD4	1	0.83	ENSG00000069696	11:637293-640706
CASP2	1	0.82	ENSG00000106144	7:142985308-143004789

target/gene	Brain_Amygdala	Brain_Anterior_cingulate_cortex_BA24	Brain_Caudate	Brain_Nucleus_accumbens	Brain_Putamen	Brain_Cerebellar_Hemisphere	Brain_Cerebellum	Brain_Cortex	Brain_Frontal_Cortex_BA9	Brain_Hippocampus	Brain_Hypothalamus	Brain_Spinal_cord_cervical_C1	Brain_Substantia_nigra	Nerve_Tibial
DRD2														-0.8
STK33		1		1		2.8	1.7							1.1
HRH1														2.5
HTR1A								2.6						
CYP2D6	-1.7	-1.5	-2	-1.9	-2.2	-1.4	-1.1	-1.6	-1.5	-1.3	-1.8	-1.6	-2.6	-1.6
ALDH1A1	2.2													-0.2
GFER			1.3	2.3				2.3						
ADRB2														-2
CASP6		2.7	0.7	0.4	0.7	1	0.9							
ABCB1									-0.2		2.2			
CACNA1H														
ESR1														
CHRM3														
BCHE											-1.7			1.9
CYP3A4														
CHRM2														
CHRM1														
BLM														
MITF														
CHRM4														
JUN														
IGF1			-0.4			-1.7								
UBE2N														
SCN5A					1.1									
SLC6A4														-0.5
KCNQ2														
HRH4														
NFE2L2											-1			
DRD4					1.4		0.5		0.2		1.5			-0.3
CASP2														

— antagonist/inhibitor/negative modulator — partial agonist — agonist/activator/positive modulator — modulator — undefined bioactivity

Supplementary Figure 15: R06AX – Other antihistamines for systemic use

drug	$-\log_{10}(p)$
ACRIVASTINE	1.82
AZATADINE	1.6
TERFENADINE	1.55
ASTEMIZOLE	1.41
RUPATADINE	1.38
EBASTINE	1.21
CYPROHEPTADINE	1.19
LORATADINE	1.05
MIZOLASTINE	1
DESLORATADINE	0.98
ANTAZOLINE	0.9
FEXOFENADINE	0.87
PHENINDAMINE	0.75
BILASTINE	0.75
BAMIPINE	0.73
EPINASTINE	0.3
AZELASTINE	0.24
TRIPROLIDINE	0.24
KETOTIFEN	0.13

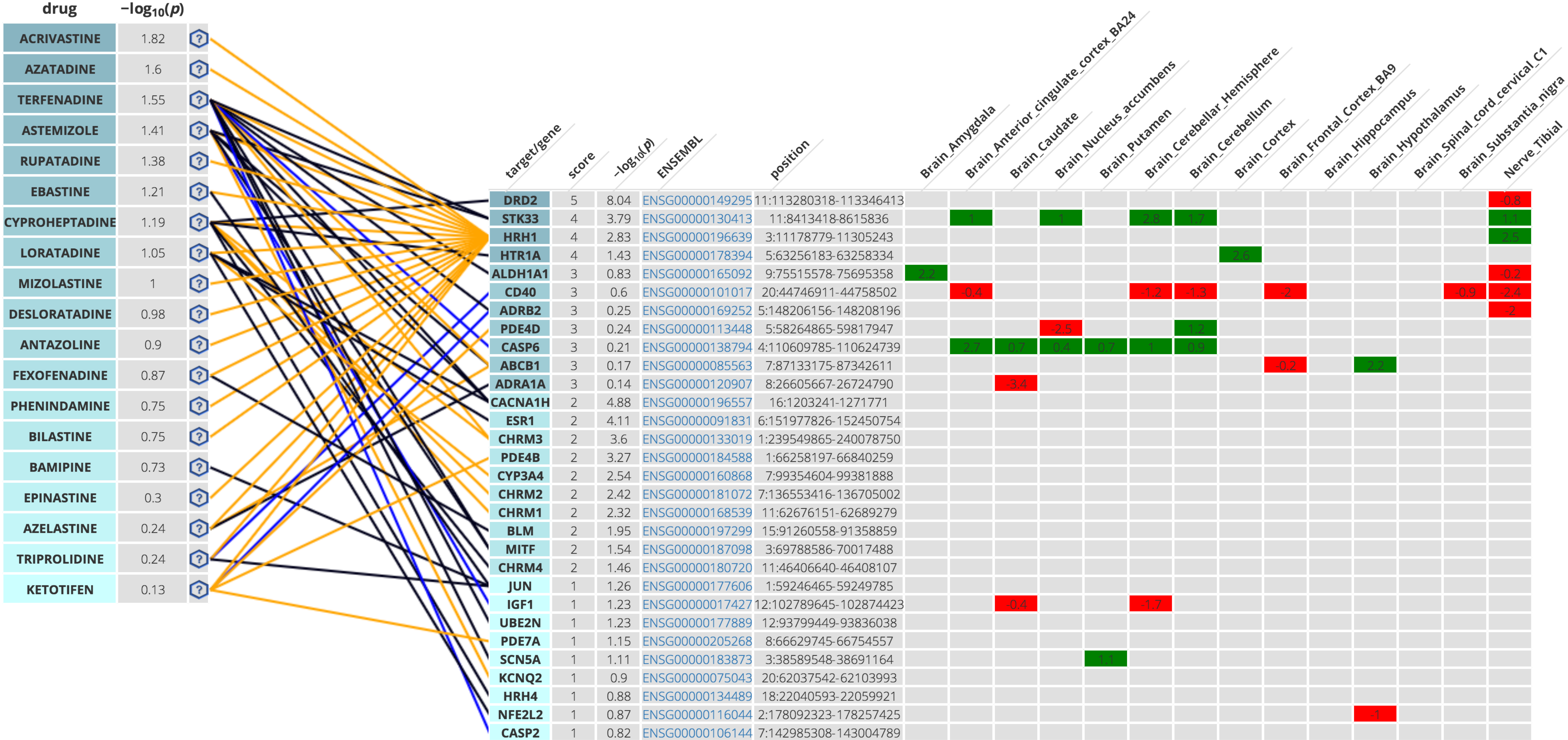

target/gene	score	$-\log_{10}(p)$	ENSEMBL	position	Brain_Amygdala	Brain_Anterior_cingulate_cortex_BA24	Brain_Caudate	Brain_Nucleus_accumbens	Brain_Putamen	Brain_Cerebellar_Hemisphere	Brain_Cerebellum	Brain_Cortex	Brain_Frontal_Cortex_BA9	Brain_Hippocampus	Brain_Hypothalamus	Brain_Spinal_cord_cervical_C1	Brain_Substantia_nigra	Nerve_Tibial
DRD2	5	8.04	ENSG00000149295	11:113280318-113346413														-0.8
STK33	4	3.79	ENSG00000130413	11:8413418-8615836		1		1		2.8	1.7							1.1
HRH1	4	2.83	ENSG00000196639	3:11178779-11305243														2.5
HTR1A	4	1.43	ENSG00000178394	5:63256183-63258334								2.6						
ALDH1A1	3	0.83	ENSG00000165092	9:75515578-75695358	2.2													-0.2
CD40	3	0.6	ENSG00000101017	20:44746911-44758502		-0.4				-1.2	-1.3		-2				-0.9	-2.4
ADRB2	3	0.25	ENSG00000169252	5:148206156-148208196														-2
PDE4D	3	0.24	ENSG00000113448	5:58264865-59817947				-2.5			1.2							
CASP6	3	0.21	ENSG00000138794	4:110609785-110624739		2.7	0.7	0.4	0.7	1	0.9							
ABCB1	3	0.17	ENSG00000085563	7:87133175-87342611									-0.2		2.2			
ADRA1A	3	0.14	ENSG00000120907	8:26605667-26724790			-3.4											
CACNA1H	2	4.88	ENSG00000196557	16:1203241-1271771														
ESR1	2	4.11	ENSG00000091831	6:151977826-152450754														
CHRM3	2	3.6	ENSG00000133019	1:239549865-240078750														
PDE4B	2	3.27	ENSG00000184588	1:66258197-66840259														
CYP3A4	2	2.54	ENSG00000160868	7:99354604-99381888														
CHRM2	2	2.42	ENSG00000181072	7:136553416-136705002														
CHRM1	2	2.32	ENSG00000168539	11:62676151-62689279														
BLM	2	1.95	ENSG00000197299	15:91260558-91358859														
MITF	2	1.54	ENSG00000187098	3:69788586-70017488														
CHRM4	2	1.46	ENSG00000180720	11:46406640-46408107														
JUN	1	1.26	ENSG00000177606	1:59246465-59249785														
IGF1	1	1.23	ENSG00000017427	12:102789645-102874423			-0.4			1.7								
UBE2N	1	1.23	ENSG00000177889	12:93799449-93836038														
PDE7A	1	1.15	ENSG00000205268	8:66629745-66754557														
SCN5A	1	1.11	ENSG00000183873	3:38589548-38691164					1.1									
KCNQ2	1	0.9	ENSG00000075043	20:62037542-62103993														
HRH4	1	0.88	ENSG00000134489	18:22040593-22059921														
NFE2L2	1	0.87	ENSG00000116044	2:178092323-178257425											-1			
CASP2	1	0.82	ENSG00000106144	7:142985308-143004789														

— antagonist/inhibitor/negative modulator — partial agonist — agonist/activator/positive modulator — modulator — undefined bioactivity
